# Extracellular Nucleic Acids Present in the *Candida albicans* Biofilm Trigger the Release of Neutrophil Extracellular Traps

**DOI:** 10.3389/fcimb.2021.681030

**Published:** 2021-05-26

**Authors:** Magdalena Smolarz, Marcin Zawrotniak, Dorota Satala, Maria Rapala-Kozik

**Affiliations:** Department of Comparative Biochemistry and Bioanalytics, Faculty of Biochemistry, Biophysics and Biotechnology, Jagiellonian University, Krakow, Poland

**Keywords:** neutrophil extracellular traps (NETs), *Candida albicans*, biofilms, extracellular nucleic acids, extracellular matrix (ECM)

## Abstract

Neutrophils, the first line of the host’s defense, use a variety of antimicrobial mechanisms to fight invading pathogens. One of the most crucial is the production of neutrophil extracellular traps (NETs) in the process called NETosis. The unique structure of NETs effectively inhibits the spread of pathogens and ensures their exposure to a high concentration of NET-embedded antimicrobial compounds. NETosis strategy is often used by the host to defend against fungal infection caused by *Candida albicans*. In immunocompromised patients, this microorganism is responsible for developing systemic fungal infections (candidiasis). This is correlated with the use of a vast array of virulence factors, leading to the acquisition of specific resistance to host defense factors and available drug therapies. One of the most important features favoring the development of drug resistance is a *C. albicans* ability to form biofilms that protect fungal cells mainly through the production of an extracellular matrix (ECM). Among the main ECM-building macromolecules extracellular nucleic acids have been identified and their role is probably associated with the stbilization of the biofilm structure. The complex interactions of immune cells with the thick ECM layer, comprising the first line of contact between these cells and the biofilm structure, are still poorly understood. Therefore, the current studies aimed to assess the release of extracellular nucleic acids by *C. albicans* strains at different stages of biofilm formation, and to determine the role of these molecules in triggering the NETosis. We showed for the first time that fungal nucleic acids, purified directly from mature *C. albicans* biofilm structure or obtained from the whole fungal cells, have the potential to induce NET release *in vitro*. In this study, we considered the involvement of TLR8 and TLR9 in NETosis activation. We showed that DNA and RNA molecules initiated the production of reactive oxygen species (ROS) by activation of the NADPH oxidase complex, essential for ROS-dependent NETosis. Furthermore, analysis of the cell migration showed that the nucleic acids located in the extracellular space surrounding the biofilm may be also effective chemotactic factors, driving the dynamic migration of human neutrophils to the site of ongoing fungal infection.

## Introduction

*Candida albicans* is a widespread eukaryotic microorganism that colonizes diverse niches in the human body (Soll et al., 1991). Many immune system disorders contribute to the loss of balance between the host's immune system and yeast, causing the development of a systemic fungal infection – candidiasis.

The ability of *C. albicans* to cause difficult-to-treat infections depends on the use of many fungal virulence factors. The most important strategies used by *C. albicans* include the production of proteins enabling adhesion to host tissue surfaces, in particular, adhesins of Als (agglutinin-like sequence) and Hwp (hyphal wall protein) protein families, the secretion of a large group of the aspartyl proteases (Saps) (Nobile et al., 2006; Naglik et al., 2008; Mayer et al., 2013; Zawrotniak et al., 2017; Rapala-Kozik et al., 2018), as well as the production of toxin—candidalysin (Naglik et al., 2019). Another virulence mechanism used by *C. albicans* is a morphological transition between a typical yeast-like form, facilitating the pathogen spread within the body, to a more virulent hyphal form, ensuring the invasion into the host tissues (Saville et al., 2003). From a perspective of the development of difficult-to-control yeast infections, the most important strategy is the formation of complex, multi-species three-dimensional structures called biofilms. Biofilms can be formed on a variety of biotic and abiotic surfaces and provide several benefits, among which the most important is the increase of drug resistance and a reduction of sensitivity to changes in environmental conditions (Taff et al., 2013). Biofilm formation by *C. albicans* is a long-term and multistage process. In the first phase, yeast cells attach to the surface due to the exposure of adhesins and change the morphological form (Cavalheiro and Teixeira, 2018). The next stage is characterized by the intensive production of a highly hydrated gel-like extracellular matrix (ECM). The process of mature biofilm formation is followed by a dispersion phase, in which budding daughter cells are released to subsequently colonize distant niches (Blankenship and Mitchell, 2006; Cavalheiro and Teixeira, 2018). The thick layer of ECM creates a physical barrier that protects *C. albicans* against chemical agents (e.g., antimycotics) and the host's immune system. The ECM components also provide additional adhesion (Hawser et al., 1998). In the case of *Candida* biofilms, ECM consists of many polymeric substances among which more than 460 functional proteins [55% wt/wt], mannan and glucan complexes (25% [wt/wt]), lipids (15% [wt/wt]) and DNA (5% [wt/wt]) have been identified (Martins et al., 2010; Zarnowski et al., 2014).

The presence of nucleic acids in the extracellular space is a phenomenon relatively well described in the case of prokaryotic biofilms. Bacterial extracellular DNA (eDNA) is involved in horizontal gene transfer (HGT) (Molin and Tolker-Nielsen, 2003). However, *in vitro* studies have shown that eDNA molecules, due to unique properties such as high viscosity and negative electrostatic charge, perform functions that are not related to the transmission of genetic information. Research has shown that eDNA molecules enhance bacterial adhesion and can additionally stabilize the structure of the biofilm (Whitchurch et al., 2002). Besides, it has been shown that eDNA can act as a chelator, binding divalent ions, e.g., magnesium (Mulcahy et al., 2008).

The first evidence for the presence of eDNA in biofilms formed by *C. albicans* was presented in 2010 (Martins et al., 2010). Its contribution to maintaining the mature *C. albicans* biofilm integrity was documented by an observation that the biomass of biofilm developed for 48 hours in the presence of deoxyribonuclease I (DNase I) was reduced by about 40%. While adding even a small amount of exogenous DNA resulted in the formation of a dense cell network (Sapaar et al., 2014). The presence of eDNA also inhibited the penetration of biofilm by antimycotics, in particular amphotericin B, and thus contributed to the increased biofilm’s drug resistance (Martins et al., 2012). Subsequent studies showed that eDNA of *C. albicans* consists mainly of random non-coding sequences, and the only exon found was a fragment encoding a cyclophilin-type peptidyl-prolyl *cis*-*trans*-isomerase (CPR3) (Zarnowski et al., 2014).

The mechanism of extracellular nucleic acid release by *C. albicans* has not been thoroughly explained so far, but it has been shown that this process can be associated with the activity of chitinases – a group of hydrolases involved in the remodeling of the cell wall during the formation of the hyphal form of the cells (Rajendran et al., 2014). Yeast cells also transport some macromolecules in extracellular vesicles (EV). Fungal EVs are often equipped with proteins, lipids, glucans, and pigments (Da Silva et al., 2015). Besides, EV released by *C. albicans* transport short (<200 bp) non-coding RNA fragments, among which micro RNA-like sequences (miRNAs) have been identified (Da Silva et al., 2015).

Neutrophils (polymorphonuclear leukocytes, PMNs) belong to the first line of defense of the host's innate immune system against a broad spectrum of invading pathogens. The recognition of pathogens by PMN leads to the activation of several fighting strategies – phagocytosis, degranulation, or the generation of neutrophil extracellular traps (NETs) in a process called NETosis (Brinkmann et al., 2004). NETs are made of decondensed chromatin fibers decorated with numerous proteins derived from azurophilic granules (in particular neutrophil elastase, myeloperoxidase, and cathepsin G), secondary and tertiary granules (e.g. lactoferrin, gelatinase), and histones (Urban et al., 2009). The unique structure of NETs allows neutrophils to immobilize pathogens and ensures the exposure to high concentrations of numerous biocidal agents (Brinkmann et al., 2004; Urban et al., 2009). There are several types of NETosis, and the choice of pathway depends on the stimulants and the receptor involved. The best-known mechanism to date is the “suicidal” NETosis, a process induced about 3 hours after pathogen recognition (Pilsczek et al., 2010). In this type of NETosis, reactive oxygen species (ROS) generation by NADPH oxidase plays a crucial role. The NET release can also occur through rapid mechanisms – ROS-independent mechanism, called “vital” NETosis (Rochael et al., 2015) and calcium ionophores-induced NETosis (Douda et al., 2015). Infections caused by small-size pathogens are effectively eradicated by phagocytosis. In infections caused by microorganisms such as *C. albicans*, which can undergo a morphological yeast-to-hyphae transition and additionally form complex biofilms, the removal of intruders via phagocytosis is relatively inefficient (Branzk et al., 2015). In the case of such infections, the preferred mechanism that effectively controls the fungal invasion is the NETosis (Urban et al., 2006; Branzk et al., 2015). In various *Candida* species, several molecules are involved in the activation of NET formation. It has been shown that fungal cell wall components such as mannans and β-glucans, as well as secreted aspartyl proteases, and even molecules engaged in the quorum-sensing (farnesol) can be involved in the induction of NET release (Byrd et al., 2013; Zawrotniak et al., 2017; Zawrotniak et al., 2019).

However, the interaction of neutrophils with the ECM layer surrounding the *C. albicans* biofilm has not been well studied, especially in terms of the role of extracellular nucleic acids. The recognition of nucleic acids of pathogenic origin by cells of the immune system is possible due to the presence of various classes of pattern recognition receptors (PRRs) located in the cytoplasm or within membranous intracellular structures called endosomes (Barrat et al., 2016). Among them, the following receptors have been distinguished: RIG-I-like (RLR), NOD-like (NLR), AIM2-like (ALR), Toll-like (TLR), cGAS, and IFI16 (Ishikawa et al., 2009; Kawasaki and Kawai, 2014; Barrat et al., 2016; Khan et al., 2019).

The direct role of nucleic acids derived from human and pathogenic cells in activating the NETosis process is poorly understood. Most published studies have mainly focused on the role of RNA molecules in this process. The first work that assigned RNA a role of potential NETosis activator concerned the activation of neutrophils by the genetic material derived from the HIV-1 retrovirus (Saitoh et al., 2012). The role of bacterial genomic RNA in the activation of ROS-dependent NETosis was demonstrated in 2017 (Rodriguez-Rodrigues et al., 2017). NETosis activation by RNA derived from human cells has also been observed. Studies performed on the psoriasis model showed that, unlike bacterial RNA, human RNA induced NETosis only when complexed with the positively charged LL-37 peptide, which was important for the translocation of RNA molecules to endosomes (Herster et al., 2020). Such an autoimmune neutrophil response was only observed for RNA-LL37 complexes, while the DNA molecules interacting with this peptide did not exert a similar. It was suggested that this process may depend on the activation of one of the intracellular receptors, particularly TLR8 that recognizes ssRNA. In contrast to nuclear DNA, it was shown that mitochondrial DNA (mtDNA) molecules released during necrosis may be a potential NETosis activator (Liu et al., 2019; Singel et al., 2019). Structurally, this DNA is similar to pathogenic genetic material because it is characterized by the presence of numerous unmethylated CpG sequences and a circular shape. A preliminary study showed that mtDNA-activated NETosis is mediated by TLR9 and cGAS receptors (Liu et al., 2019). The only work that indirectly suggested the involvement of eDNA located in the ECM of the bacterial biofilm in NETosis induction was a finding that the degradation of the nucleic acid-rich matrix surrounding the biofilm of *P. aeruginosa* with DNase I inhibited the ability of biofilms to activate the neutrophil pro-inflammatory response and significantly reduced the amount of released NETs (Fuxman Bass et al., 2010).

The significance of extracellular nucleic acids derived from the eukaryotic pathogen for the induction of NETosis has not yet been demonstrated. Therefore, the current study aimed to present a role of extracellular nucleic acids, isolated from *C. albicans* biofilm and whole cells, in the induction of the NETosis process and verify their chemotactic properties.

## Materials and Methods

### Yeast Strains and Culture

*C. albicans* strains ATCC 10231 and SC5413 were grown in YPD medium (1% yeast extract, 2% soybean peptone, and 2% glucose) at 30°C for 16 h on an orbital rotary shaker MaxQ 6000 (180 rpm) (Thermo Fisher Scientific, Waltham, MA, USA) to the stationary phase. Cells numbers were determined by optical density (OD) measurements at 600 nm, assuming that OD_600_=1 value corresponds to 3x10^7^ cells per 1 ml.

### Biofilm Formation 

Biofilm formation was performed using *C. albicans* cells from the stationary growth phase. The cells were centrifuged (3000 rpm, 5 min), and washed twice with sterile PBS. The suspensions of *Candida* cells (10^5^ - 10^7^ cells/ml) in 100 μl of RPMI 1640 medium w/o phenol red (Biowest, Nuaille, France) were added into microplate wells, with a high-binding surface (Corning Corporation, Corning, NY), and the biofilms were formed for 48 h at 37°C under aerobic conditions in an incubator (Galaxy 170R, Eppendorf, Hamburg, Germany).

To isolate eDNA and eRNA, the cells were grown in 20 ml of RPMI 1640 medium, starting from 1 ml of cells from the stationary phase. Cells were incubated under aerobic conditions for 48 h at 37°C on an orbital shaker in a 200 ml Erlenmeyer flask (170 rpm).

To observe biofilm growth over time, *C. albicans* (10^4^ cells/ml) were cultured for 24 h at 37°C under aerobic conditions in an incubator (Galaxy 170R, Eppendorf, Hamburg, Germany) and then for a further 24 h in a microscope incubator (Leica, Wetzlar, Germany).

### Detection of Extracellular Nucleic Acids

To identify the presence of eDNA and eRNA in the biofilm matrix, selected fluorescent dyes were used: Sytox Green (Molecular Probes, Eugene, OR) at a final concentration of 1 μM for DNA and RNA staining, Quant-iT PicoGreen (Invitrogen, Waltham, MA) at a 200-fold dilution of concentrated dye for dsDNA staining, and SYTO RNA Select (Invitrogen, Waltham, MA, USA) at a concentration of 500 nM for RNA identification. Biofilms were imaged using AE31E (Motic, Barcelona, Spain), IX73 (Olympus, Tokyo, Japan), and DMI6000B (Leica, Wetzlar, Germany) fluorescence microscopes. Additionally, the presence of eDNA and eRNA in the biofilm was confirmed by nuclease treatments. After 48 hours of biofilm culturing in 96-well plate (10^5^ cells/ml), DNase I (a final concentration of 20 U/ml; Thermo Scientific, Waltham, MA) or RNase A (a final concentration of 0.2 mg/ml; Thermo Scientific, Waltham, MA) in a reaction buffer containing MgCl_2_ (Thermo Scientific, Waltham, MA) were added to RPMI 1640 medium. The incubation with nucleases was carried out for 1.5 h. After the digestion, extracellular nucleic acids were stained with Sytox Green and the fluorescence was measured using a Biotek Synergy H1 microplate reader (excitation: 485 nm, emission: 528 nm). Biofilms not treated with nucleases served as a reference.

### Isolation and Purification of Extracellular Nucleic Acids

To isolate eDNA/eRNA, we applied a slightly modified method successfully used for the isolation of fungal multifunctional proteins, deposited on the surface of the fungal cells (Zawrotniak et al., 2017). *C. albicans* cells that formed biofilms on the surface of Erlenmeyer flask walls were gently scratched, and the cell pellet was suspended in 100 mM Tris-HCl buffer with 0.9% NaCl pH 7.4, and then washed: once with 100 mM Tris-HCl buffer with 0.9% NaCl pH 7.4, and twice 50 mM Tris-HCl buffer pH 7.5. The solutions of cell wall-deposited components were prepared by treatment of biofilm-forming cells with β-1,3-glucanase (Lyticase from *Arthrobacter luteus*; Sigma-Aldrich, St. Louis, MO). Lyticase (1250 U per 1 g of biofilm wet mass) was added to the cell suspension in 2 ml of 50 mM Tris buffer, pH 7.5 containing 40 mM β-mercaptoethanol and protease inhibitor cocktail (Roche, Penzberg, Germany). Cells were incubated for 2 h at 37°C with gentle shaking (170 rpm). Trypan Blue staining was used to verify that at least 95% of the *C. albicans* cells remained intact after this treatment. After centrifugation of the cells (1500 x g, 3 min), the supernatant, containing surface-located material, was transferred into new Eppendorf tubes, and a series of centrifugations (3000 x g, 3 min; 6000 x g, 6 min; 10000 x g, 10 min) were performed to remove insoluble material. The supernatant was dialyzed against 50 mM Tris-HCl pH 7.5 for two days. Nucleic acids were separated from the cell wall proteins (CWP) by ion-exchange chromatography (IEC) on MonoQ-Sepharose column (GE Healthcare/Pharmacia, Uppsala, Sweden) equilibrated in 50 mM Tris-HCl buffer, pH 7.5. The CWP fractions were eluted using a 10 min gradient of NaCl (0 - 0.4 M) in 50 mM Tris-HCl buffer, pH 7.5, and the fractions of the nucleic acids were eluted during 10 min gradient of NaCl (0.4 – 1 M) in 50 mM Tris-HCl buffer, pH 8.5 ml (procedure based on Zawrotniak et al., 2017). The fractions containing the highest concentration of nucleic acids, identified by the measurement of OD_260nm_, were collected and dialyzed against 20 mM MOPS buffer with 350 mM NaCl (pH 6.25). For further separation of eDNA from eRNA molecules, the second chromatographic step on the MonoQ column was used. In the elution process, a NaCl gradient (0.3 – 1 M) in 20 mM MOPS (pH 6.25) optimized based on the work of Jahn et al. (1991) was used. Finally, the buffer in the collected samples was exchanged to sterile PBS using the desalting column (PD-10, GE Healthcare). Sample purity was assessed using absorbance measurement in the range from 240 nm to 300 nm with a Biotek Synergy H1 microplate reader, which showed the absence of protein and organic reagent impurities.

### Genomic DNA Isolation

*C. albicans* cells from an overnight culture in YPD were placed in an Eppendorf tube (2x10^8^ cells/tube), centrifuged, and washed twice with PBS buffer. Then, sterile glass beads (425-600 μm) and 400 μl of reagent containing phenol:chloroform:isoamyl alcohol (25:24:1) and 400 μl of yeast lysis buffer (2% Triton X-100; 10% SDS; 5M NaCl; 1M Tris-HCl pH 8; 0.5M EDTA) were added to the cell pellet. The yeast cell homogenization process was carried out using the Bertin Precellys Evolution cell homogenizer. The homogenate was centrifuged (10 min, 12000 x g, 4°C). Then equal volume of chloroform:isoamyl alcohol (24:1) mixture was added to the aqueous phase of homogenate and centrifuged (10 min, 12000 x g, 4°C). To precipitate DNA, 1/10 volume of 0.3 M sodium acetate (pH 5.5) and 2 volumes of 96% ethanol were added to the aqueous phase, then the samples were incubated overnight at -20°C. After incubation, samples were centrifuged (15 min, 12000 x g, 4°C). The white precipitate, containing DNA, was washed twice with 70% ethanol and dried, then dissolved in 100 μl of nuclease-free water. Sample purity was assessed using a sample absorbance measurement in the range from 240 nm to 300 nm with a Biotek Synergy H1 microplate reader.

### Total RNA Isolation

Sterile glass beads (diameter: 425-600 μm) and 800 μl of TRI-Reagent were added to the washed cell pellet obtained as above mentioned. Similarly, to DNA isolation, the yeast cell homogenization process was carried out. 160 μl chloroform was added to the homogenate and incubated for 30-minutes at 4°C, followed by centrifugation of the samples (10,000 x g, 15 min, 4°C). An equal volume of isopropanol was added to the aqueous phase and the samples were placed at -20°C overnight. The samples were then centrifuged (10,000 x g, 15 min, 4°C) and the supernatant was gently removed. The pellet was suspended in 70% ethanol and centrifuged after a few minutes of incubation (10,000 x g, 15 min, 4°C). The resulting white RNA pellet was washed twice with 70% ethanol and dried, then dissolved in 100 μl of nuclease-free water. RNA concentration and purity were determined analogously to DNA acquisition.

### Neutrophil Isolation

Human polymorphonuclear cells (PMNs) were isolated from EDTA-treated (5 mM) whole-blood samples obtained from healthy donors via the Regional Blood Donation Center (Cracow, Poland). The entire isolation process was carried out at room temperature, while all reagents used were preheated to 37°C. To separate morphotic elements from the plasma, the blood samples (20 ml) were centrifuged at 420 x g for 20 min. Then 2/3 of the upper phase containing the plasma was discarded and the bottom layer containing leukocytes and erythrocytes was restored to the original volume of 20 ml with PBS and thoroughly mixed by inverting the tube. The cell suspension was carefully layered on the lymphocyte separation medium (Biowest, Nuaille, France). After centrifugation (420 x g for 30 min.), the low-density fractions were discarded. To the lower phase, containing erythrocytes and granulocytes, 2 volumes of 1% polyvinyl alcohol were added. The separation of erythrocytes from granulocytes was carried out for 20 min, after which the upper phase, containing granulocytes, was collected into a new tube and centrifuged at 420 x g for 6 minutes. For additional purification of isolated granulocytes, the cells were suspended in 1 ml of Red Blood Lysis Buffer (Roche, Penzberg, Germany) and then washed with PBS. The granulocyte pellet was suspended in 1 ml RPMI 1640 medium w/o phenol red (Biowest, Nuaille, France). The number of cells was determined using a Bürker chamber. The neutrophil purity was assessed routinely by forward- and side-scatter flow cytometric analyses. The applied isolation method yields a >95% pure population of the cells. Due to the short life of neutrophils, all experiments were carried out immediately after isolation.

### Test of Neutrophil Viability

Immediately after the isolation process, viability analysis of isolated neutrophils was performed, which was based on staining cells with propidium iodide (PI) and fluorescein isothiocyanate (FITC)-labeled Annexin V (AnV) (Dead Cell Apoptosis Kit with Annexin V-FITC and PI, Invitrogen, Carlsbad, CA, USA). Cells (1x10^6^) were washed three times with PBS and stained with PI and AnV for 15 minutes, according to the supplier’s instruction. Cell viability assessment was performed using flow cytometry (LSR Fortressa, BD, San Jose, CA, USA).

### NET Visualization and Quantification

Neutrophils (10^6^ cells/ml) were seeded in the well of the 96-wells black microplate (Greiner Bio-One, Frickenhausen, Germany), in 100 µl of RPMI 1640 and allowed to settle for 20 min at 37°C, 5% CO_2_. Then, cells were washed twice with PBS, and yeast DNA and RNA samples at variable concentration, prepared in RPMI 1640 medium, were added. Unstimulated neutrophils and neutrophils treated with 25 nM of PMA (Sigma-Aldrich) served as negative and positive controls, respectively. The stimulation of neutrophils was carried out for 3 hours at 37°C, 5% CO_2_. After incubation, the stimulants were removed, and the cells were washed gently with PBS. For the nucleic acid quantification, micrococcal nuclease (MNase) at a concentration of 1 U/ml (Roche, Penzberg, Germany) was added, and the neutrophils were incubated at 37^o^C for 20 min to release NETs into the supernatant. The collected supernatants were centrifuged and stained with Sytox Green fluorescence dye (final concentration 1 μM). 50 μl of each sample was transferred into a 96-well microplate to measure the fluorescence (excitation: 495 nm, emission: 525 nm) using the microplate reader.

For fluorescence microscopy analyzes, stimulated neutrophils were gently washed with PBS and fixed with 3.6% paraformaldehyde for 10 min at room temperature. Then, primary antibodies (1:200): anti-neutrophil elastase (NE; Abcam, Cambridge, UK) and anti-myeloperoxidase (MPO; Abcam, Cambridge, UK), diluted in PBS buffer with 0.5% BSA, were added and the samples were incubated overnight at 4°C. After incubation, neutrophils were gently washed twice with PBS, and secondary anti-rabbit (1:500; Abcam, Cambridge, UK) antibodies were added. The plates were incubated for 1 h at 37°C and cells were washed twice with PBS. Additionally, NETs were labeled with the Sytox Green dye (final concentration 1 μM) and visualized using a fluorescence microscope, as previously described.

### Analysis of ROS Production

The production of reactive oxygen species (ROS) was analyzed using the method based on the fluorescence of oxidized dihydrorhodamine 123 (DHR 123; Invitrogen, Waltham, MA). Neutrophils (10^6^ cells/ml) were added in 100 µl RPMI 1640 and allowed to settle for 20 min at 37° C, 5% CO_2_ in the wells of 96-well microplate. Neutrophils were washed with PBS, and nonfluorescent DHR 123 was added to each well at a final concentration of 10 μM and incubated for 10 minutes. Then, 10 µl of yeast nucleic acids (a final concentration of 0.6 µg/ml) were added to cells. Untreated neutrophils were used as a negative control, and cells, stimulated with 25 nM PMA, as a positive control. The fluorescence of oxidized DHR 123 was recorded immediately after adding stimulants, for one hour with minimal integration time, using a microplate reader.

### NADPH Oxidase Inhibition

To assess the role of ROS in NETosis induction via yeast DNA and RNA, freshly isolated neutrophils were preincubated for 30 min at 37°C in RPMI 1640 medium with 10 μM DPI (Sigma-Aldrich), a commonly used NADPH oxidase inhibitor. After incubation, cells were washed twice with PBS and stimulated as mentioned above. Untreated neutrophils were used as control.

### Inhibition of Endocytosis

The role of endocytosis in the induction of NETosis was determined by the use of cytochalasin D, a compound inhibiting actin microfilament polymerization. Freshly isolated neutrophils were preincubated for 30 min at 37°C in RPMI 1640 medium with 5 μM cytochalasin D (CytD, Sigma-Aldrich). After incubation, the inhibitor was removed, cells were washed twice with PBS and stimulated with yeast nucleic acids as described above. Untreated neutrophils were used as control.

### Analysis of TLR8 Receptor Activation

The presence of the TLR8 (CD288) receptor in human neutrophils was verified using flow cytometry. For this purpose, freshly isolated neutrophils were fixed with 3.6% paraformaldehyde. Then, the cells were permeabilized with Triton X-100, and after washing twice with sterile PBS, the cells were incubated overnight at 4°C with phycoerythrin-conjugated (PE) anti-human TLR8 antibodies (BioLegend, San Diego, CA, USA). Identification of yeast RNA binding receptors was carried out using CU-CPT9a molecule (InvivoGen, San Diego, CA, USA), which prevents the activation of TLR8. In these experiments, neutrophils (10^6^ cells/ml, 100 µl RPMI-1640) settled on the microplate were stimulated for 3 h with yeast RNA (a final concentration of 0.5-1.2 μg/ml) and PMA (positive control; a final concentration of 25 nM) diluted in a solution containing CU-CPT9a (a final concentration of 100 nM). Neutrophils treated by the same concentration of CU-CPT9a but without stimulants served as a negative control.

### Chemotaxis Analysis

The chemotaxis of neutrophils in the presence of yeast DNA and RNA was evaluated by the 24-well chemotaxis chamber technique (Transwell®-Clear inserts, Corning, NY, USA). 100 μl of neutrophil suspension (10^7^ cells/ml) were applied to the inserts (3 µm pores) in PBS buffer. Samples of DNA and RNA (0.5 μg/ml), fMLP (10 µM; used as a positive control), and PBS (negative control) were placed in the lower compartment. The chambers were incubated at 37°C in 5% CO_2_ atmosphere for 30 min. The cells that migrated into the lower compartment of the chamber were fixed and quantified.

### Statistical Analysis

Statistical analysis was performed with the GraphPad Prism 8 software (GraphPad Software, CA, USA). The results are presented as a mean value with a standard error of the mean (SEM). To assess significance between groups, unpaired t-test or one-way ANOVA with Dunnett's multiple comparisons post-hoc test were performed. The results were considered statistically significant at p-value <0.05 (*p ≤ 0.0332, **p < 0.0021, ***p < 0.0002, ****p < 0.0001) or not statistically significant (ns) for p > 0.1234.

## Results

### Extracellular Nucleic Acids Are Present in the ECM Biofilm Formed by *C. albicans*

**Figure 1:**
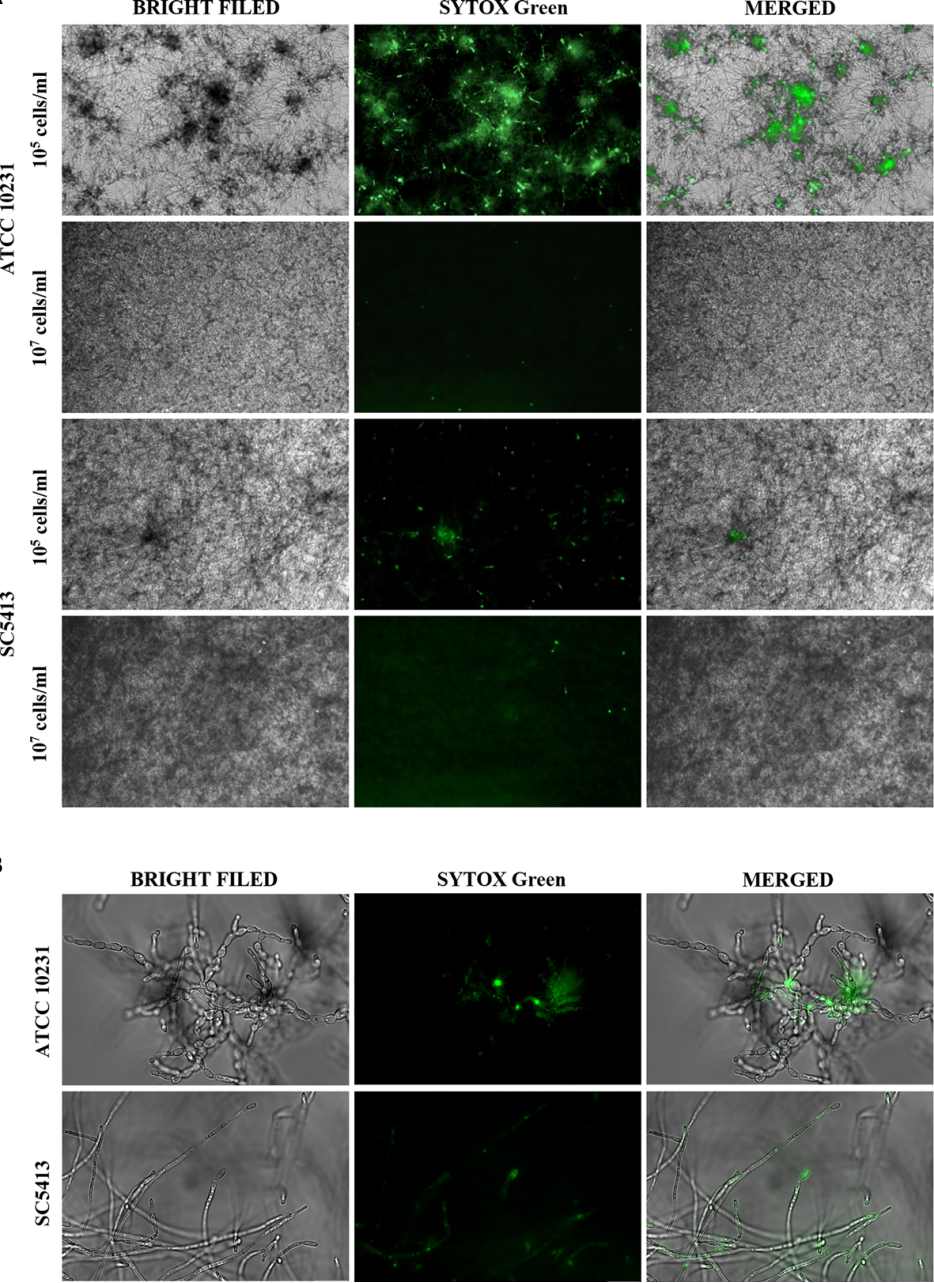
Extracellular nucleic acids presence in the ECM of *C. albicans* biofilm. **(A)** 10^5^ cells/ml or 10^7^ cells/ml of *C. albicans* were placed in a 96-well plate in 100 μl RPMI 1640 and cultured aerobically for 48 hours at 37°C. The formed biofilms were stained with Sytox Green (a final concentration of 1 μM) and imaged using fluorescence microscopy in the green channel and transmitted light (AE31E, Motic microscope). **(B)** *C. albicans* (ATCC 10231, SC5413) cells present in hyphal form in 48 hours-biofilm (10^5^ cells/ml) were visualized with an Olympus IX73 microscope.

We compared the morphology of biofilms and nucleic acid exposure for two *C. albicans* strains: ATCC 10231 and SC5413. Our observation suggested that the release of extracellular nucleic acids depends on the initial cell number and may be correlated with cell morphology (**Figure 1A**). The standardization of culture conditions showed that a relatively small initial cell count (10^5^ cells/ml) is crucial for the efficient production of extracellular nucleic acids. For this number of cells, induction of filamentous growth was observed for both strains. Increasing the cell density (up to 10^7^ cells/ml) inhibited the formation of hyphae and decreased the release of nucleic acids. However, the obtained results showed significant differences between these strains, both in the morphology of the formed biofilms and the ability to nucleic acid release For cells of SC5413, the formation of typical long and thin hyphae covering the entire plate's surface was observed (**Figure 1B**). In contrast, the ATCC 10231 cells formed rather pseudohyphae, characterized by the presence of constrictions at the septal site. ATCC 10231 biofilm formed characteristic dense clusters in which significant amounts of nucleic acids were located.

**Figure 2:**
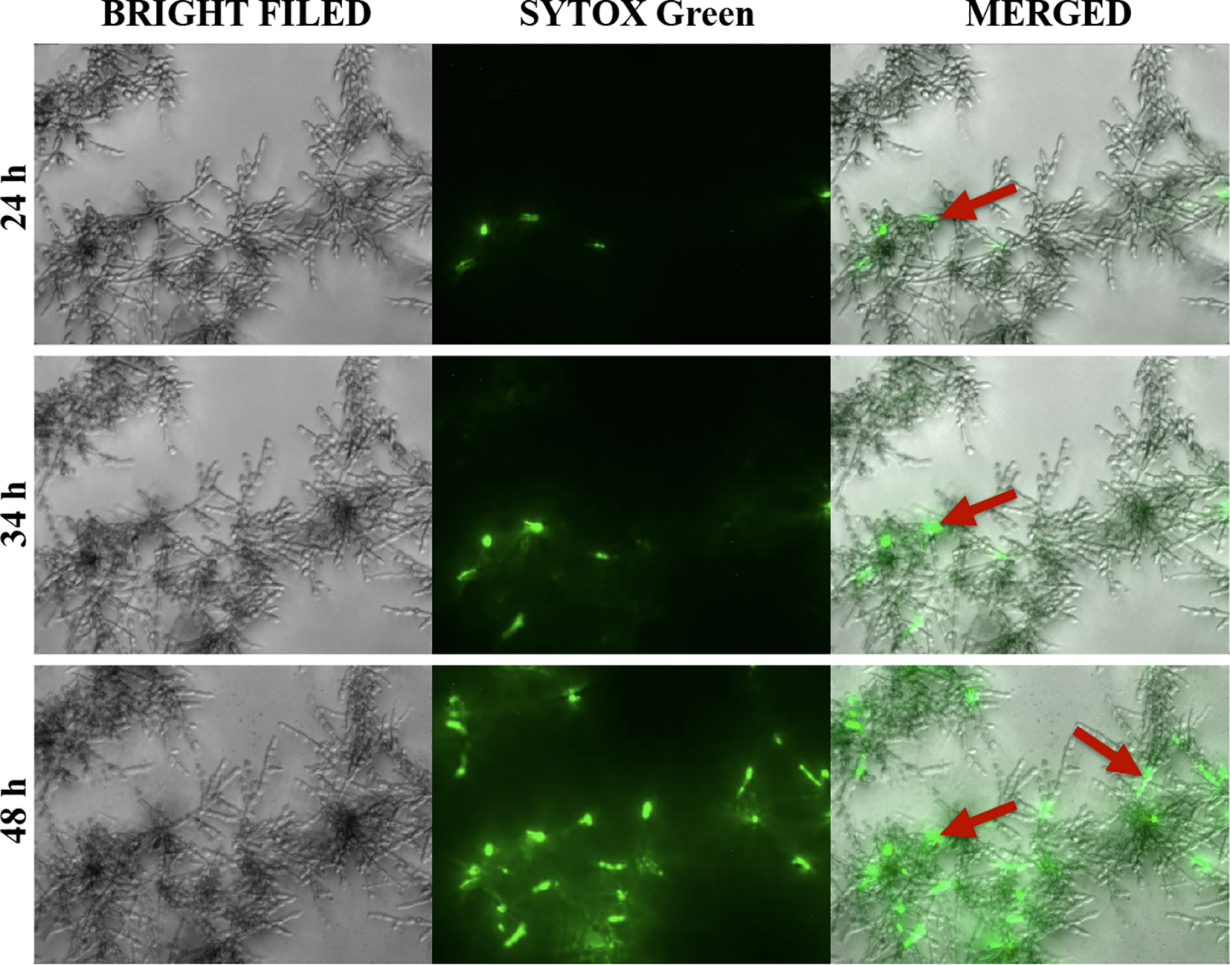
Release of extracellular nucleic acids during the formation of biofilms by *C. albicans* (ATCC 10231). Cells (10^4^/ml) were placed in a 96-well plate, in 100 μl RPMI 1640 and incubated at 37°C for 24 hours. Then, the Sytox Green dye was added to the biofilm formed, and microscopic observation was continued for a further 24-hour (DMI6000B microscope, Leica).

To determine at which stage of biofilm formation the extracellular nucleic acids are released, we carried out the microscopic observation of biofilm growth progress and found that the release of nucleic acids started at least after 24 hours of biofilm formation, and their exposure increased over the next hours (34–48 h). Clusters of nucleic acids were a feature of mature, minimum 48-hour biofilms. The effect of nucleic acid release over time was shown at the three selected time points and marked with arrows (**Figure 2**).

**Figure 3:**
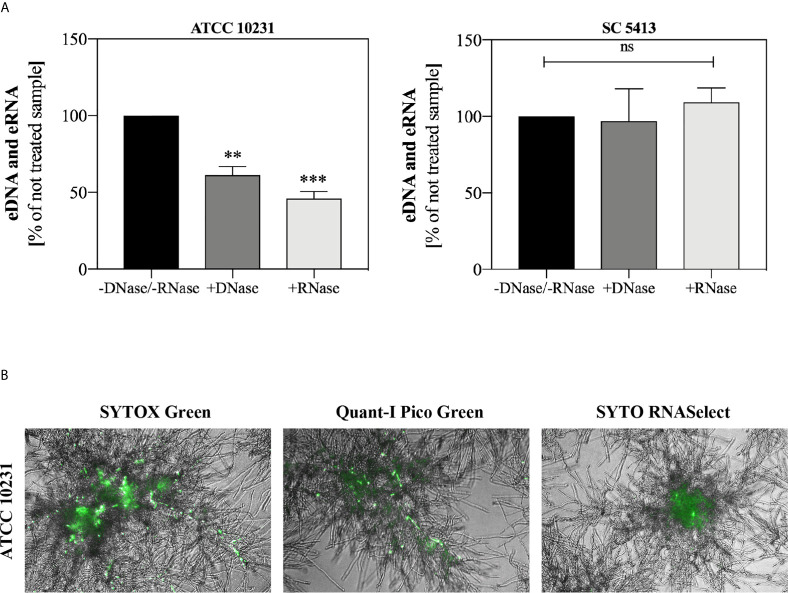
Extracellular nucleic acids in *C. albicans* biofilm. **(A)** *C. albicans* biofilms (ATCC 10231 and SC5413; 10^5^ cells/ml) were cultured at 37°C in 100 µl RPMI 1640 medium for 48 hours. Then biofilms were incubated with DNase I (20 U/ml) and RNase A (0.5 mg/ml) for 1.5 h. After incubation, the fluorescence of the Sytox Green dye (a final concentration of 1 μM) was measured using a microplate reader (Biotek, Synergy H1). The results represent the mean fluorescence value of three replicates after 48 hours of culture. ANOVA and Dunnett’s multiple comparisons post-test was used. The results were considered statistically significant at p < 0.05 (**p ≤ 0.0021, ***p ≤ 0.0002) or not statistically significant (ns) for p > 0.1234. **(B)** *C. albicans* cells (ATCC 10231, 10^5^ cells/ml) were placed in three separate wells of 96-well high-binding plate and cultured at 37°C for 48 hours under aerobic conditions in 100 μl RPMI 1640 medium. The biofilms were then stained with fluorescent dyes that selectively bind the nucleic acids (Sytox Green, 1 μM; Quant-iT PicoGreen, 200-fold diluted; Syto RNASelect, 500 nM) and visualized in transmitted light and green fluorescence (AE31E, Motic).

Additionally, we conducted an experiment involving the addition of DNase I and RNase A to 48-hour biofilms. After 1.5 h of enzymatic degradation of eDNA/eRNA, the biofilms were stained with Sytox Green and the fluorescence of the biofilm was measured. Biofilms not treated with nucleases (-DNase/-RNase) served as negative controls. The results showing the percentage of fluorescence of control biofilms (without DNase/RNase) are presented in **Figure 3A**. Our observations indicated that ATCC 10231 strain formed a biofilm containing a significant amount of extracellular nucleic acids, with the domination of eRNA, in an opposite to biofilms formed by strain SC5413 where no significant effect of nucleases on the eDNA or eRNA was observed. For further identification of both types of nucleic acids in ATCC 10231 biofilm, we used selective dyes, characterized by an increased affinity for dsDNA (Quant-iT PicoGreen, Invitrogen) and RNA (SYTO RNASelect, Invitrogen) (**Figure 3B**).

### *C. albicans* Extracellular Nucleic Acids Trigger NET Release

**Figure 4:**
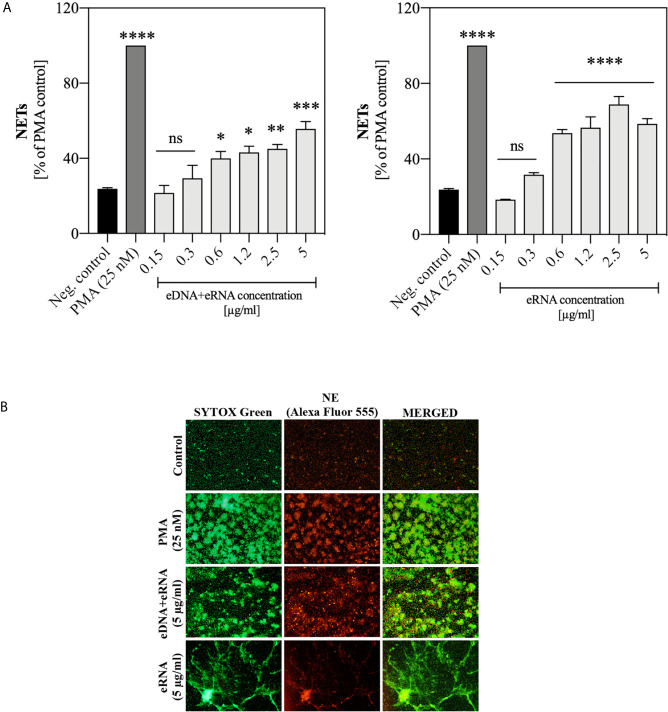
Neutrophil stimulation with eRNA and eDNA. Neutrophils (10^6^ cells/ml) were incubated for 3 hours with chromatographically purified extracellular nucleic acids (0.15 - 5 μg/ml). Unstimulated cells served as a negative control and the positive control was represented by the response of neutrophils to 25 nM PMA. **(A)** The released NETs were digested with MNase (a final concentration of 1 U/ml) and the collected samples were stained with Sytox Green (1 μM). Results are shown as percent fluorescence of the positive control (PMA) ± standard error of the mean (SEM) for two replicates. ANOVA with Dunnett’s multiple comparisons post-hoc test was used. The results were considered statistically significant at p < 0.05 (*p ≤ 0.0332, **p ≤ 0.0021, ***p ≤ 0.0002, ****p < 0.0001 or not statistically significant (ns) for p > 0.1234. **(B)** To confirm the presence of NET structures, after stimulation neutrophils were fixed with 3.6% paraformaldehyde and then incubated overnight at 4°C with rabbit primary antibodies anti-NE (200-fold diluted in PBS with 0.5% BSA). The released NETs were detected with anti-rabbit secondary antibodies, conjugated to the Alexa Fluor 555 (500-fold diluted in PBS with 0.5% BSA) and Sytox Green dye (1 μM). NETs were visualized using fluorescence microscopy (AE31E, Motic).

The first experiments, aimed at a preliminary assessment of the possible activation of the NETosis process in human neutrophils by fungal nucleic acids, were carried out using samples purified by IEC (**Supplementary Figure 1**) and containing pure eRNA or a mixture of eDNA and eRNA. We analyzed the neutrophil responses to the extracellular nucleic acid concentration in a range of 0.15 μg/ml to 5 μg/ml. Statistically, significant NET production was observed above 0.6 μg/ml for both types of samples. In this concentration range, for samples containing a mixture of nucleic acids, we observed the generation of NETs at the level of 40-60% of the positive control, but in the case of pure eRNA fraction, the response ranged from 50-70%. Representative results are shown in **Figure 4A**. To identify the NET structures, the Sytox Green dye and primary antibodies against the characteristic NET marker - neutrophil elastase (NE), were used. The results confirmed that the DNA was released by neutrophils in the NETosis process, and not as a result of accidental neutrophil cell rupture or nonspecific binding of fungal eDNA/eRNA to the cells (**Figure 4B**).

**Figure 5:**
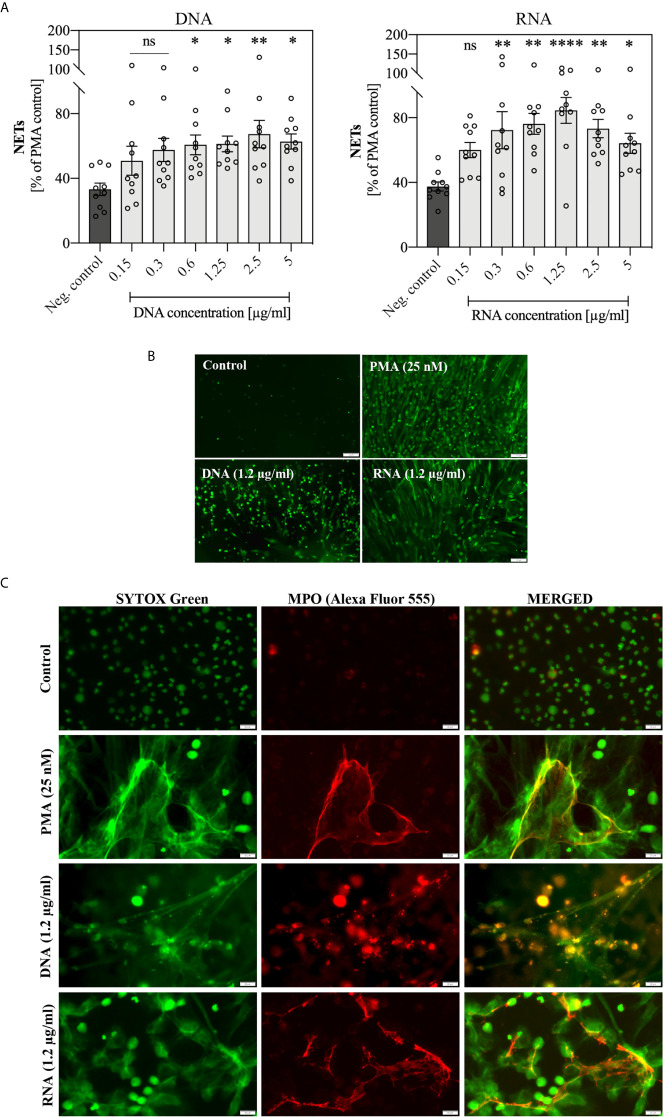
Stimulation of neutrophils with yeast nucleic acid samples (DNA, RNA). Neutrophils (10^6^ cells/ml) were incubated at 37°C for 3 hours with yeast DNA and RNA samples in a concentration range of 0.15 – 5 μg/ml. **(A)** Released NETs were quantified in the supernatant after 15 minutes of treatment with MNase (a final concentration of 1 U/ml). Data represent a comparison of NET release for 10 independent experiments ± SEM. ANOVA with Dunnett’s multiple comparisons post-hoc test was used (*p ≤ 0.0332, **p ≤ 0.0021, ****p ≤ 0.0001, or not statistically significant (ns) for p > 0.1234). **(B)** After stimulation, unfixed NETs were stained with Sytox Green (1 μM) and visualized using Motic AE31E fluorescence microscopy. **(C)** After stimulation, cells were fixed 3.6% paraformaldehyde, rabbit primary antibodies anti-MPO (200-diluted) were added to fixed neutrophils, stimulated previously with 1.2 μg/ml DNA or RNA, and incubated overnight at 4°C. The released NETs were detected with anti-rabbit secondary antibodies conjugated to the Alexa Fluor 555 (500-fold diluted) or Sytox Green dye (1 μM) and was visualized using Olympus IX73 fluorescence microscope.

Because obtaining a pure fraction of eDNA in IEC was inefficient, and the response of cells to DNA and RNA molecules may follow different signaling paths, in subsequent experiments we used both types of nucleic acids, isolated from the whole *C. albicans* cells using methods ensuring their high purity. For the application of pure DNA and RNA samples, a series of 10 independent experiments with healthy donor samples were performed involving the stimulation of neutrophils with a wide range of nucleic acid concentrations. The results, comparing the neutrophil response to yeast DNA and RNA, are shown in **Figures 5A, B**. The presence of NET structures was confirmed using antibodies that recognized MPO (**Figure 5C**).

The obtained results showed that the optimal concentration at which NET release in response to genomic DNA and total RNA was observed, was within a range of 0.3 – 5 μg/ml. Moreover, the activation of NETosis by RNA molecules was more efficient than by DNA. Independent experiments showed that the neutrophil response to DNA and RNA was characterized by relatively high variability between healthy donors.

### Kinetics of NET Release Upon Contact With Yeast DNA and RNA

**Figure 6:**
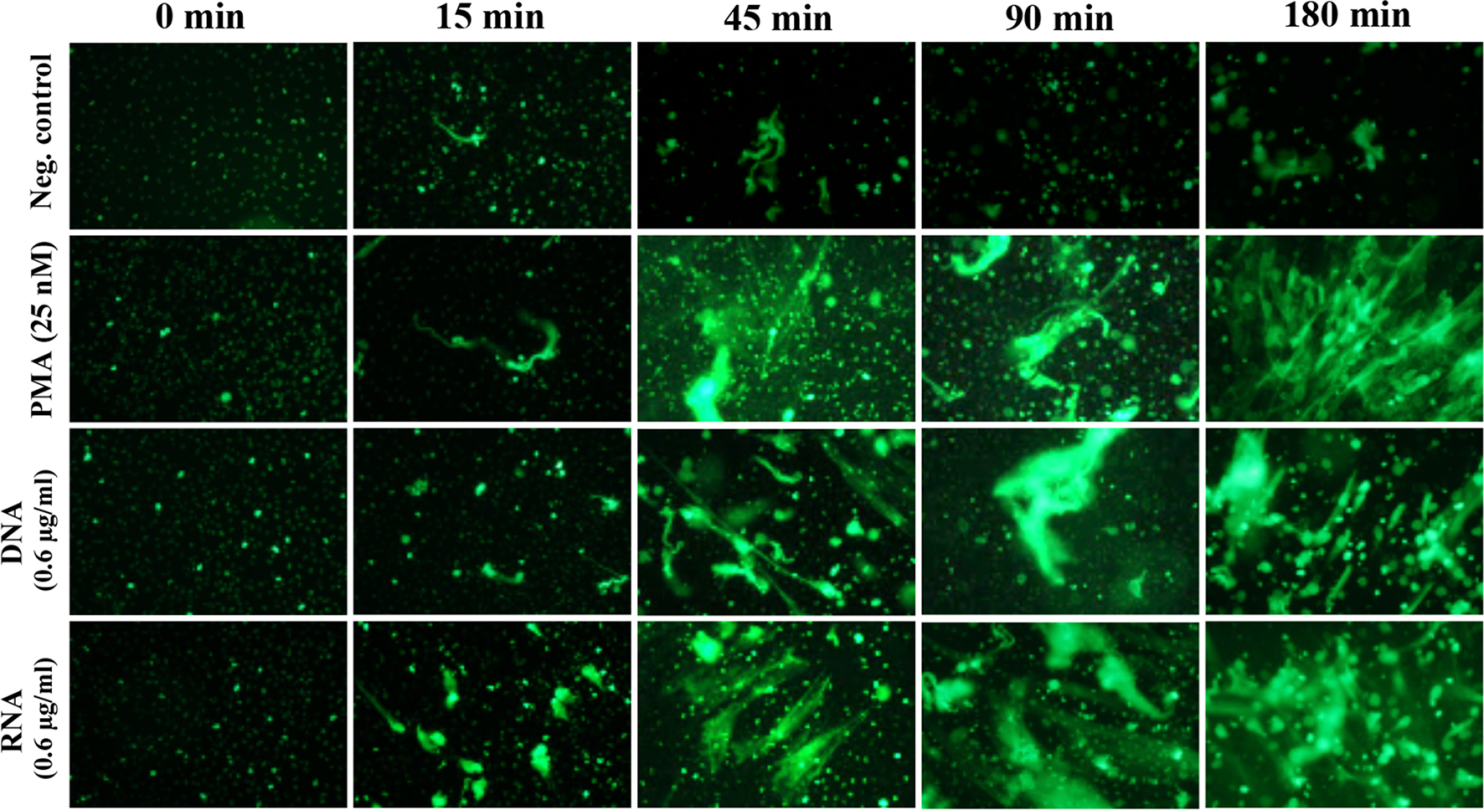
Kinetics of NET release. Neutrophils (10^6^ cells/ml; 100 μl per well) were incubated for 2 hours with DNA and RNA isolated from the planktonic form of *C. albicans* (0.6 μg/ml). Released NETs were fixed at specific time points, then stained with Sytox Green dye, and imaged using fluorescence microscopy (as described above).

The activation of the NETosis mechanism, depending on the type of stimulating factors, took up to 3 hours. Relatively fast responses were induced by chemical compounds, which freely diffuse across the membrane and directly activate signal pathway mediators. The fast generation of NETs was also characteristic of the vital and calcium ionophores-induced NETosis mechanisms of NETosis (Douda et al., 2015; Rochael et al., 2015). To analyze the kinetics of the NETosis triggered by yeast DNA and RNA, neutrophils (10^6^ cells/ml; 100 μl per well, in separate series for each time point) were incubated with the selected concentration of nucleic acids (0.6 μg/ml) up to 3 hours, and NET production was determined at each time point, using sample staining with Sytox Green dye (**Figure 6**). The response of neutrophils to nucleic acid treatment started after 45 minutes, similar to the results observed for neutrophils activated with PMA.

### Yeast DNA and RNA Induction of NETosis Is ROS-Dependent 

**Figure 7:**
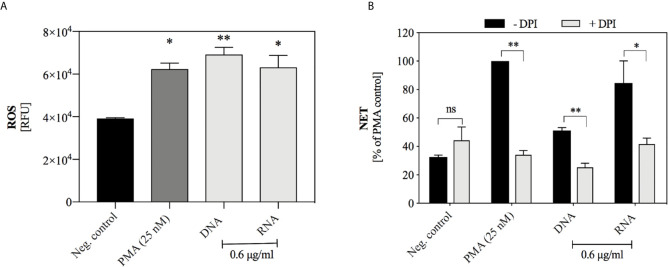
The role of ROS-dependent pathway in the NET release. **(A)** DHR 123 (10 μM) was added to neutrophils (10^6^ cells/ml) suspended in 100 µl RPMI 1640 and after 10 minutes of incubation, DNA or RNA samples at the final concentration of 0.6 µg/ml were added. Untreated neutrophils served as a negative control, and cells stimulated with 25 nM PMA represented a positive control. The fluorescence of oxidized DHR 123 was measured for one hour with minimal kinetic interval, using a BioTek Synergy H1 microplate reader. Graphs present the values of the fluorescence increase ± SEM (n=2). **(B)** Neutrophils (10^6^/ml cells; 100 μl per well) with inhibited NADPH oxidase activity (+ DPI, 10 μM) were incubated with yeast DNA and RNA (0.6 μg/ml) for 3 hours. Neutrophils not treated with inhibitor but stimulated with DNA or RNA served as a control (- DPI). Results are presented as a percentage of response to PMA (-DPI) ± SEM for n=2. ANOVA with Dunnett’s multiple comparisons post-hoc test was used. The differences considered statistically significant at p < 0.05 (*p ≤ 0.0332, **p ≤ 0.0021) or not statistically significant (ns) for p > 0.1234.

The kinetics of NET release triggered by nucleic acids suggested that the mechanism of NETosis activation may be similar to the process initiated by PMA treatment of neutrophils and may depend on the production of ROS. To confirm the participation of ROS in this process, we used neutrophil staining with dihydrorhodamine 123 (DHR 123) prior to the addition of nucleic acids, as this dye passively diffuses across membranes, and after oxidation localizes to the mitochondria. The obtained results (**Figure 7A**) indicated that both yeast DNA and RNA at a concentration of 0.6 μg/ml induced the oxidation of DHR 123, at a level comparable to PMA treatment. Additionally, freshly isolated neutrophils were preincubated for 30 minutes with NADPH oxidase inhibitor (DPI, 10 μM) before the addition of yeast DNA and RNA samples (0.6 μg/ml) (**Figure 7B**). The inhibition of NADPH oxidase resulted in the reduction of NET production to the level of the negative control, suggesting that the induction of NETosis by nucleic acids used a ROS-dependent pathway.

### Nucleic Acid Endocytosis Is a Prerequisite for NETosis

**Figure 8:**
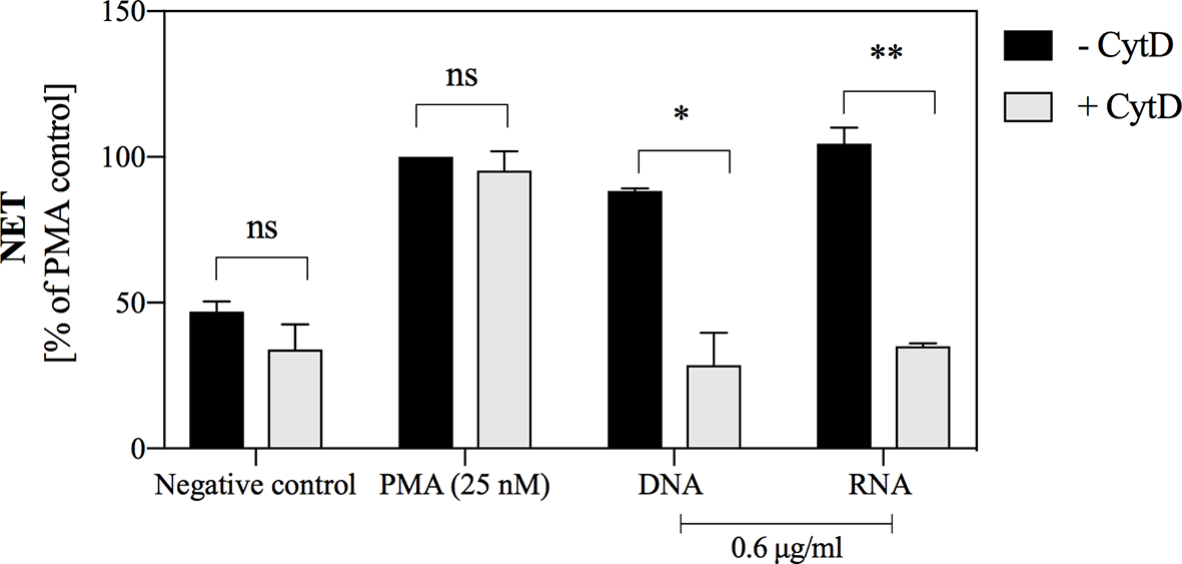
The role of endocytosis in the induction of NETosis, stimulated by yeast DNA and RNA. Neutrophils with inhibited endocytosis ability by the use of cytochalasin D (10^6^ cells/ml; 100 μl per well) were incubated with yeast DNA and RNA at a concentration of 0.6 μg/ml for 3 hours. Neutrophils with the active cytoskeleton (- CytD) served as control. NETs were quantified in supernatants after MNase treatment (a final concentration of 1 U/ml). Results are presented as a percentage of response relatively to that to PMA (- CytD) for n=2. Unpaired t-test was used to compare differences between the NETosis intensity of -CytD and + CytD cells. The differences considered statistically significant at p <0.05 (*p ≤ 0.0332, **p ≤ 0.0021) or not statistically significant (ns) for p > 0.1234.

The use of cytochalasin D (CytD), an inhibitor of actin fiber polymerization, allowed to indirectly determine whether endocytosis of yeast nucleic acids is a necessary step to induce the NETosis process. A quantitative analysis (**Figure 8**) showed that the use of cytochalasin D inhibited the NETosis process induced by nucleic acids but did not affect the overall capacity for NETosis, as evidenced by the lack of inhibitory effect during neutrophil stimulation with PMA. The findings indicated that yeast nucleic acids induce NET formation via the endocytosis-dependent pathway.

### TLR8 Receptor Is Involved in the Activation of Yeast RNA-Induced NETosis 

**Figure 9:**
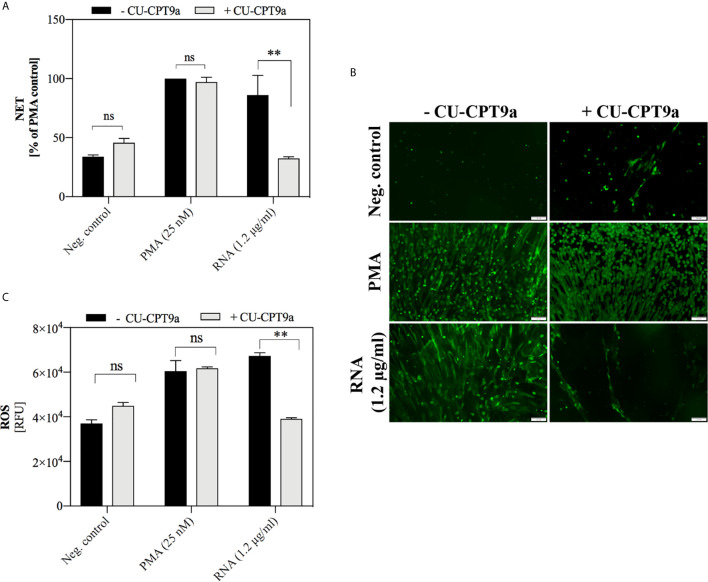
The role of TLR8 receptor in NETosis activation by yeast RNA. **(A)** Neutrophils (10^6^ cells/ml; 100 μl per well) were stimulated at 37°C under 5% CO_2_ atmosphere for 3 h with yeast RNA (1.2 μg/ml) or PMA (positive control; a final concentration of 25 nM) diluted in CU-CPT9a solution (100 nM). RPMI 1640 medium containing the same concentration of inhibitor served as the negative control. Released NETs were stained with Sytox Green (1 μM) and quantified in supernatants after MNase treatment (a final concentration of 1 U/ml). **(B)** Released NETs were stained with Sytox Green dye and imaged using Olympus IX73 fluorescence microscope. **(C)** To neutrophils (10^6^ cells/ml) suspended in inhibitor (100 nM) and dihydrorhodamine 123 (10 μM) solutions, RNA was added at a final concentration of 0.6 µg/ml. ROS production was measured for one hour using a BioTek Synergy H1 microplate reader. Unpaired t-test was used to compare differences between the NETosis intensity and ROS production of -CU-CPT9a and +CU-CPT9a. The differences considered statistically significant at p <0.05 (**p ≤ 0.0021) or not statistically significant (ns) for p > 0.1234.

The well-known receptor that recognizes RNA molecules is TLR8 (CD288), located within endosomes (Barton and Kagan, 2009). Its level in the tested neutrophils was verified by flow cytometry, each time before the main experiment (**Supplementary Figure 2**). To assess whether yeast RNA-induced NETosis is related to TLR8 receptor activation, we used the CU-CPT9a molecule, which by binding to the TLR8 dimer, stabilizes the receptor in its resting state and antagonizes further conformational changes caused by the stimulating factors (Zhang et al., 2018). Our data showed that the induction of NETosis by yeast RNA was lowered in the presence of CU-CPT9a in the medium, supporting the conclusion of TLR8 participation in this process (**Figures 9A, B**). However, the antagonistic effect of CU-CPT9a was not observed during stimulation of the cells with PMA, which confirmed that the applied compound did not contribute to the loss of the ability of cells to NETosis. We also checked whether the applied TLR8 antagonist could affect the RNA-mediated production of ROS (**Figure 9C**). The results showed that the presence of CU-CPT9a lowered ROS production to the negative control level, confirming the role of TLR8 in the recognition of yeast RNA.

Similar analyzes were undertaken to determine the receptor involved in the activation of NETosis by yeast DNA. However, the use of a short synthetic suppressive oligonucleotide (CpG ODNs), identified as TLR9 antagonist (Lenert, 2010), did not produce conclusive results (**Supplementary Figure 3**). The role of the receptor involved in the recognition of yeast DNA remains to be elucidated.

### Nucleic Acids of *C. albicans* Are Strong Chemotactic Factors

**Figure 10:**
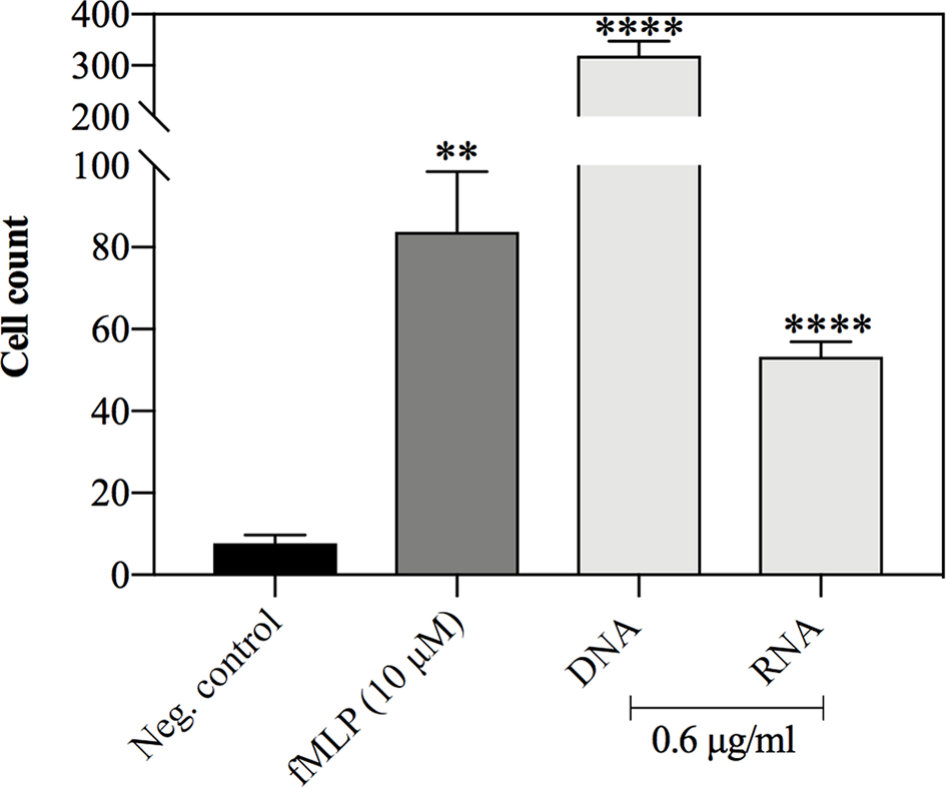
Neutrophil chemotaxis in response to yeast DNA and RNA. Neutrophils (10^7^ cells/ml, 1 ml per well) were incubated for 30 minutes in chemotaxis chambers with yeast DNA, RNA (0.6 μg/ml), and fMLP (10 μM), which served as a positive control. Negative control represented the cells treated with PBS. After the incubation, the neutrophils that were found at the bottom of the plate were fixed and counted. The data present the mean cell count for 4 independent repetitions. Unpaired t-test was used to compare differences between means. The results were considered statistically significant at p <0.05 (**p ≤ 0.0021, ****p > 0.0001) or not statistically significant (ns) for p > 0.1234.

Neutrophil movements in the presence of stimulating factors were studied using dedicated chambers, containing trans-well inserts. The chemotactic agents (yeast DNA and RNA at final concentration 0.6 μg/ml) were added to the bottom of the chamber, while neutrophils were placed within the inserts, separated by a porous membrane (3 μm). As the negative control, PBS was used, whereas the positive control was represented by the responses of neutrophils to 10 μM solution of fMLP, a characteristic bacterial peptide, being a well-known chemotactic agent. The number of cells that passed through the membrane according to the factor gradient was counted after one hour of treatment. The obtained results showed that both yeast nucleic acids stimulated neutrophil chemotaxis, but yeast DNA molecules are particularly efficient chemoattractants for neutrophils, comparing to yeast RNA (**Figure 10**).

## Discussion

Recent research has allowed for the characterization of the main *C. albicans* virulence factors, owing to which this fungal pathogen can successfully colonize various niches in the host's organism and defend against its immune system. One of the important strategies favoring the development of life-threatening fungal infections is the formation of biofilms, that, mainly due to the presence of a dense layer of the extracellular matrix, provide a protective environment for fungi, increase their adhesion, and contribute to drug resistance (Cavalheiro and Teixeira, 2018).

The components of the ECM include nucleic acids, which, apart from their basic function of genetic information storage, are also able to perform other roles in the extracellular space. An example is the phenomenon of NETosis, where the extracellular DNA of neutrophils is involved in the host’s defense. However, microbial nucleic acids may also play important functions in the extracellular space within bacterial or fungal biofilms (Whitchurch et al., 2002; Okshevsky and Meyer, 2015; Rostami et al., 2016). The studies carried out so far have focused mainly on the analysis of the role of extracellular nucleic acids present in the ECM of monospecies bacterial biofilms, while little attention has been paid to the analysis of this phenomenon in eukaryotic and mixed biofilms.

Therefore, the first stage of our research was to unequivocally confirm the reports of other researchers indicating the presence of extracellular nucleic acids in the matrix of biofilms formed by the pathogenic yeast-like fungus *C. albicans* (Martins et al., 2010; Martins et al., 2012; Rajendran et al., 2014). We conducted a series of optimizations aimed at determining *in vitro* conditions favorable for the release of measurable amounts of extracellular nucleic acid but we keep in mind that the standardization is performed on the artificial surface. The standardization concerned a wild-type *C. albicans* strain ATCC 10231 and a highly virulent strain SC5413. Fluorescence microscopy analyzes showed significant differences in nucleic acid-releasing ability between those strains. These differences may be correlated with the morphology of biofilm-forming cells. In the case of strain ATCC 10231, we observed cell growth mainly in the form of pseudohyphae, while the SC5314 strain forms typical long, thin hyphae which promotes increased tissue penetration and increases virulence (Thewes et al., 2008). It can be assumed that the pseudohyphal form may favor the release of eDNA and eRNA, which can form a gel-like structure of the ECM, as was proposed for the biofilm of *P. aeruginosa* (Seviour et al., 2019). Such conception could support the finding that the ATCC10231 biofilm was more resistant to substances affecting cell membrane integrity (Lupetti et al., 2002).

Moreover, the observations are consistent with the results obtained by Rajendran et al. (2014) who reported a positive correlation between the expression of genes associated with the change of morphological form (encoding chitinases and adhesin HWP1) and the efficient release of eDNA by *C. albicans*. Furthermore, the fact that obtaining a biofilm surrounded by eDNA and eRNA is only possible for cultures with a relatively small initial cell number, confirmed the correlation with cell morphology, which is regulated by quorum sensing molecules i.e farnesol and its derivatives (Hornby et al., 2001; Oh et al., 2001; Alem et al., 2006). In the case of a greater initial number of cells, the formation of hyphae is inhibited, and according to our results, is correlated with a reduced amount of extracellular nucleic acids.

Our time-lapse analysis demonstrated that the release of detectable amounts of nucleic acids occurs after about 48 hours, in contrast to bacterial biofilms where the appearance of eDNA was reported at the early stages of biofilm formation (Whitchurch et al., 2002). A similar observation was presented by Martins et al. (2010) who suggested that extracellular nucleic acids are components that ensure the stability of a mature *C. albicans* biofilm and are not essential for the early stage of yeast adhesion. Such a result was also presented in the case of biofilms formed by another pathogenic fungus *Aspergillus fumigatus* (Rajendran et al., 2014).

In studies published so far, where the analysis of *Candida* biofilms structure has been conducted, it has been mentioned that mainly eDNA molecules appear among the components of the protein-lipid matrix (Martins et al., 2010; Martins et al., 2012; Rajendran et al., 2014; Sapaar et al., 2014). Our results presented that, in addition to eDNA, the biofilm structure contains also a significant amount of eRNA. Interesting studies were carried out by Da Silva et al. (2015) who showed that short fragments (<250 nucleotides) of *C. albicans* RNA may be transported by MVs, supporting our finding of a significant amount of eRNA in ATCC 10231 biofilm. In turn, the results obtained in the case of bacterial biofilm indicated that DNA and RNA molecules may form complexes by Watson-Crick interactions and nonspecific bonds (Seviour et al., 2019). Such structures may favor the formation of a dense ECM, stabilizing the bacterial biofilm. These results suggest that a similar situation may occur in the case of biofilms formed by *C. albicans.*

Recent studies have focused mainly on proving a possibility of induction of NETosis as a result of contact with molecules exposed on the *C. albicans* cells surface, which include mainly β-glucans (Brogden et al., 2014), mannans, and through the secreted virulence factors such as aspartyl proteases (Zawrotniak et al., 2017) or by the action of the fungal quorum-sensing molecule – farnesol (Zawrotniak et al., 2019). Many previous studies aimed to determine the mechanism of neutrophil responses to planktonic *C. albicans* form. However, the biofilm structure, due to the presence of a dense layer of ECM, forming a physical barrier, masks pathogen’s cells, and partially protect them against attack by the host's immune system. Such impairment in NET release in contact with *C. albicans* biofilm was reported by Johnson and co-workers (Johnson et al., 2016). The possibility of inducing NET release by yeast nucleic acids has not been considered so far. Few studies, that used bacterial, viral, or human nucleic acids have suggested that in particular, RNA molecules have some potential to activate NET production (Saitoh et al., 2012; Rodriguez-Rodrigues et al., 2017; Herster et al., 2020). We revealed for the first time that nucleic acids obtained from ECM of *C. albicans* biofilm and whole yeast cells growing in planktonic form have a significant potential to activate NETosis. Our analysis indicated that the neutrophil response to fungal nucleic acids can be observed even at their low concentrations, pointing at the possible rapid activation of immune mechanisms at the site of infection. In most of our experiments, we observed that NET release in response to RNA/eRNA molecules was more effective than the response to DNA molecules. A similar observation indicating a weaker neutrophil response to human DNA than RNA (in the form of a DNA/LL37 complex) was already observed in the study of Herster et al. (2020) in a model of psoriasis.

Both NETosis and phagocytosis, on the one hand, use similar signaling pathways and the pathogen-killing arsenal, but, on the other hand, are classified as separate fighting mechanisms (Hakkim et al., 2011; Branzk et al., 2015; Manfredi et al., 2018). Analyzes suggest that NETosis may be activated as a result of ineffective phagocytosis resulting, for example, from the large size of the pathogen (Branzk et al., 2015). Considering the previous reports, we initially checked whether the endocytosis of nucleic acids is a necessary step to initiate NETosis. We showed that cytoskeleton activity is necessary for the NET release during yeast DNA- and RNA-induced NETosis but not in the case of PMA. Such a scenario may result from the intracellular location of receptors that recognize nucleic acids, and thus from the necessity of translocation of the stimulating molecule into the cell compartment. Our observations are in line with the results published by Rodriguez-Rodrigues et al. (2017) that neutrophils, stimulated with long fragments of bacterial RNA and with nystatin-inhibited endocytosis induced NETosis in a lowered yield. The situation is slightly different in the case of neutrophil responses to human RNA, which require the presence of the LL-37 peptide for recognizing, and such a complex mediates RNA internalization and further translocation into intracellular compartments (Herster et al., 2020). The contribution of receptors that may be involved in the binding of free DNA and RNA molecules on the outer side of the membrane and thus in the initiation of the internalization is still poorly understood. A possibility of exposure of TLR9 receptors on the surface of neutrophils was suggested (Lindau et al., 2013), which may be an important clue in the search for a mechanism of DNA internalization. In the case of receptors recognizing RNA, their exposure to the extracellular space has not been confirmed so far, and the possible mechanism of RNA internalization remains unknown.

Knowing that *C. albicans* DNA and RNA/eRNA have the potential to activate NETosis, we analyzed the role of two of the best-known receptors for nucleic acids expressed by human neutrophils i.e TLR8 and TLR9 (Hayashi et al., 2003; Janke et al., 2009). It is known that activation of these receptors drives the production of type I interferon and other cytokines (Alvarez-Arellano et al., 2014; Nascimento et al., 2019), but the reports indicating TLR8 involvement in activating NETosis are relatively recent. Our data obtained in an attempt to verify TLR9 activation by yeast DNA molecules, using antagonistic suppressive oligonucleotides (TTAGGG), did not bring unequivocal results. Moreover, there is no conclusive information in the literature on the relationship of any DNA recognition receptor with the induction of this process. The main concern is finding the appropriate TLR9 blocking agent. The TTAGGG sequence shows an inhibitory effect on cellular function after several hours of pre-incubation, and this is longer than expected for a NETosis occurrence. So it excludes the oligonucleotide usage in the study. The role of RNA molecules in the activation of NET production was demonstrated in the context of human and bacterial samples (Rodriguez-Rodrigues et al., 2017; Herster et al., 2020). The cited studies suggested that this process most likely depends on TLR8 activation. Our results are consistent with these observations. We demonstrated that the use of CU-CPT9 compound preventing the formation of an active TLR8 conformation, blocked *C. albicans* RNA-induced NETosis.

In further research, we have made attempts to determine the detailed mechanism underlying the activation of NETosis via *C. albicans* DNA and RNA. We checked whether the NETosis pathway is related to the production of ROS. The results indicated that both yeast DNA and RNA molecules activate the oxidative burst. These results were additionally supported by the complete inhibition of NET production after the use of a specific NADPH oxidase inhibitor. Besides, the time and course of NETosis activation by yeast nucleic acids corresponds to the response to PMA, a compound that is a classical activator of NADPH oxidase and ROS-dependent NETosis (Cox et al., 1985; Brinkmann et al., 2004). Involvement of the ROS-dependent pathway was also observed in response to bacterial RNA (Rodriguez-Rodrigues et al., 2017). Additionally, we showed that the activation of ROS production by RNA is correlated with the participation of the TLR8 receptor.

Recent studies have allowed the identification of several chemotactic factors produced by *C. albicans* cells (Gabrielli et al., 2016; Zawrotniak et al., 2017; Zawrotniak et al., 2019). We also decided to analyze the chemotactic potential of human neutrophils in response to yeast nucleic acids. The role of nucleic acid molecules as factors activating cell migration has not been well investigated so far, and the only information on this topic appeared in 2017 and concerned the response to bacterial RNA (Rodriguez-Rodrigues et al., 2017). Our results indicated a surprisingly high chemotactic potential of *C. albicans* DNA and RNA molecules. In all experiments carried out, a very dynamic migration, occurring after a few minutes of stimulation was observed. The obtained results indicated that the extracellular nucleic acids present in the ECM, surrounding the biofilm, may be an important chemotactic factor driving dynamic migration of neutrophils to the site of an ongoing fungal infection.

The results presented in this paper show for the first time that extracellular nucleic acids appearing in *C. albicans* biofilms during mature biofilm formation may be factors driving the dynamic response of human neutrophils at late stages of fungal infection.

Activation of NETosis in contact with fungal biofilm exposing eRNA/eDNA in ECM could also be supported by the antifungal treatment, such as with echinocandins. By influencing the fungal cell organization, it can augment the innate immune response of the host (Hoyer et al., 2018). A better understanding of fungal biofilm formation, composition and stability during infection progression will open new avenues for the development of effective antifungal therapies.

## Data Availability Statement

The original contributions presented in the study are included in the article/**Supplementary Material**. Further inquiries can be directed to the corresponding author.

## Author Contributions

Conceptualization: MS, MZ, and MR-K. Methodology and investigation: MS, MZ, and DS. Data analysis: MS, MZ, DS, and MR-K. Writing (original draft): MS. Writing (review and editing): all authors. Funding acquisition: MR-K. Supervision: MR-K and MZ. All authors contributed to the article and approved the submitted version.

## Funding

This work was financially supported by the National Science Centre of Poland (grant no. 2019/33/B/NZ6/02284 awarded to MR-K).

## Conflict of Interest

The authors declare that the research was conducted in the absence of any commercial or financial relationships that could be construed as a potential conflict of interest.

## References

[B1] Alem M. A. S. Oteef M. D. Y. Flowers T. H. Douglas L. J. (2006). Production of Tyrosol by *Candida Albicans* Biofilms and Its Role in Quorum Sensing and Biofilm Development? Eukaryot. Cell 5, 1770–1779. 10.1128/EC.00219-06 16980403 PMC1595342

[B2] Alvarez-Arellano L. Cortés-Reynosa P. Sánchez-Zauco N. Salazar E. Torres J. Maldonado-Bernal C. (2014). TLR9 and NF-κb are Partially Involved in Activation of Human Neutrophils by *Helicobacter Pylori* and Its Purified DNA. PLoS One 9, e101342. 10.1371/journal.pone.0101342 24987851 PMC4079333

[B3] Barrat F. J. Elkon K. B. Fitzgerald K. A. (2016). Importance of Nucleic Acid Recognition in Inflammation and Autoimmunity. Annu. Rev. Med. 67, 323–336. 10.1146/annurev-med-052814-023338 26526766

[B4] Barton G. M. Kagan J. C. (2009). A Cell Biological View of Toll-Like Receptor Function: Regulation Through Compartmentalization. Nat. Rev. Immunol. 9, 535–542. 10.1038/nri2587.A 19556980 PMC3934928

[B5] Blankenship J. R. Mitchell A. P. (2006). How to Build a Biofilm: A Fungal Perspective. Curr. Opin. Microbiol. 9, 588–594. 10.1016/j.mib.2006.10.003 17055772

[B6] Branzk N. Lubojemska A. Hardison S. E. Wang Q. Maximiliano G. Brown G. D.. (2015). Neutrophils Sense Microbial Size and Selectively Release Neutrophil Extracellular Traps in Response to Large Pathogens. Nat. Immunol. 15, 1017–1025. 10.1038/ni.2987 PMC4236687 25217981

[B7] Brinkmann V. Reichard U. Goosmann C. Fauler B. Uhlemann Y. Weiss D. S.. (2004). Neutrophil Extracellular Traps Kill Bacteria. Science 303, 1532–1535. 10.1126/science.1092385 15001782

[B8] Brogden G. Krimmling T. Adamek M. Naim H. Y. Steinhagen D. Von Köckritz-Blickwede M. (2014). The Effect of β-Glucan on Formation and Functionality of Neutrophil Extracellular Traps in Carp (*Cyprinus Carpio L.*). Dev. Comp. Immunol. 44, 280–285. 10.1016/j.dci.2014.01.003 24434196

[B9] Byrd A. S. O’Brien X. M. Johnson C. M. Lavigne L. M. Reichner J. S. (2013). An Extracellular Matrix-Based Mechanism of Rapid Neutrophil Extracellular Trap Formation in Response to C. Albicans. J. Immunol. 190, 4136–4148. 10.4049/jimmunol.1202671.An PMC3622194 23509360

[B10] Cavalheiro M. Teixeira M. C. (2018). *Candida* Biofilms: Threats, Challenges, and Promising Strategies. Front. Med. 5, 28. 10.3389/fmed.2018.00028 PMC5816785 29487851

[B11] Cox J. A. Jeng A. Y. Sharkey N. A. Blumberg P. M. Tauber A. I. (1985). Activation of the Human Neutrophil Nicotinamide Adenine Dinucleotide Phosphate (NADPH)-Oxidase by Protein Kinase C. J. Clin. Invest. 76, 1932–1938. 10.1172/JCI112190 2997297 PMC424245

[B12] Da Silva R. P. Puccia R. Rodrigues M. L. Oliveira D. L. Joffe L. S. César G. V.. (2015). Extracellular Vesicle-Mediated Export of Fungal RNA. Sci. Rep. 5, 1–12. 10.1038/srep07763 PMC5379013 25586039

[B13] Douda D. N. Khan M. A. Grasemann H. Palaniyar N. (2015). SK3 Channel and Mitochondrial ROS Mediate NADPH Oxidase-Independent NETosis Induced by Calcium Influx. Proc. Natl. Acad. Sci. U. S. A. 112, 2817–2822. 10.1073/pnas.1414055112 25730848 PMC4352781

[B14] Fuxman Bass J. I. Russo D. M. Gabelloni M. L. Geffner J. R. Giordano M. Catalano M.. (2010). Extracellular DNA: A Major Proinflammatory Component of *Pseudomonas Aeruginosa* Biofilms. J. Immunol. 184, 6386–6395. 10.4049/jimmunol.0901640 20421641

[B15] Gabrielli E. Sabbatini S. Roselletti E. Kasper L. Perito S. Hube B.. (2016). In Vivo Induction of Neutrophil Chemotaxis by Secretory Aspartyl Proteinases of *Candida Albicans* . Virulence 7, 819–825. 10.1080/21505594.2016.1184385 27127904 PMC5029300

[B16] Hakkim A. Fuchs T. A. Martinez N. E. Hess S. Prinz H. Zychlinsky A.. (2011). Activation of the Raf-MEK-ERK Pathway Is Required for Neutrophil Extracellular Trap Formation. Nat. Chem. Biol. 7, 75–77. 10.1038/nchembio.496 21170021

[B17] Hawser S. P. Baillie G. S. Douglas L. J. (1998). Production of Extracellular Matrix by *Candida Albicans* Biofilms. J. Med. Microbiol. 47, 253–256. 10.1099/00222615-47-3-253 9511830

[B18] Hayashi F. Means T. K. Luster A. D. (2003). Toll-Like Receptors Stimulate Human Neutrophil Function. Blood 102, 2660–2669. 10.1182/blood-2003-04-1078 12829592

[B19] Herster F. Bittner Z. Archer N. K. Dickhöfer S. Eisel D. Eigenbrod T.. (2020). Neutrophil Extracellular Trap-Associated RNA and LL37 Enable Self-Amplifying Inflammation in Psoriasis. Nat. Commun. 11, 105. 10.1038/s41467-019-13756-4 31913271 PMC6949246

[B20] Hornby J. M. Jensen E. C. Lisec A. D. Tasto J. Jahnke B. Shoemaker R.. (2001). Quorum Sensing in the Dimorphic Fungus *Candida Albicans* Is Mediated by Farnesol. Appl. Environ. Microbiol. 67, 2982–2992. 10.1128/AEM.67.7.2982 11425711 PMC92970

[B21] Hoyer A. R. Johnson C. J. Hoyer M. R. Kernien J. F. Nett J. E. (2018). Echinocandin Treatment of *Candida Albicans* Biofilms Enhances Neutrophil Extracellular Trap Formation. Antimicrob. Agents Chemother. 62 (9), e00797–e00718. 10.1128/AAC.00797-18 29987146 PMC6125570

[B22] Ishikawa H. Ma Z. Barber G. N. (2009). STING Regulates Intracellular DNA-Mediated, Type I Interferon-Dependent Innate Immunity. Nature 461, 788–792. 10.1038/nature08476 19776740 PMC4664154

[B23] Jahn M. J. Jahn D. Kumar A. M. Soil D. (1991). Mono Q Chromatography Permits Recycling of DNA Template and Purification of RNA Transcripts After T7 RNA Polymerase Reaction. Nucleic Acids Res. 19, 2786. 10.1093/nar/19.10.2786 1710347 PMC328209

[B24] Janke M. Poth J. Wimmenauer V. Giese T. Coch C. Barchet W.. (2009). Selective and Direct Activation of Human Neutrophils But Not Eosinophils by Toll-like Receptor 8. J. Allergy Clin. Immunol. 123, 1026–1033. 10.1016/j.jaci.2009.02.015 19361845

[B25] Johnson C. J. Cabezas-Olcoz J. Kernien J. F. Wang S. X. Beebe D. J. Huttenlocher A.. (2016). The Extracellular Matrix of *Candida Albicans* Biofilms Impairs Formation of Neutrophil Extracellular Traps. PLoS Pathog. 12, 1–23. 10.1371/journal.ppat.1005884 PMC5021349 27622514

[B26] Kawasaki T. Kawai T. (2014). Toll-Like Receptor Signaling Pathways. Front. Immunol. 5, 461. 10.3389/fimmu.2014.00461 25309543 PMC4174766

[B27] Khan S. Godfrey V. Zaki M. H. (2019). “Cytosolic Nucleic Acid Sensors in Inflammatory and Autoimmune Disorders,” in International Review of Cell and Molecular Biology (Elsevier), p. 215–253. 10.1016/bs.ircmb.2018.10.002 30798989

[B28] Lenert P. S. (2010). Classification, Mechanisms of Action, and Therapeutic Applications of Inhibitory Oligonucleotides for Toll-Like Receptors (TLR) 7 and 9. Mediators Inflamm. 2010, 986596. 10.1155/2010/986596 20490286 PMC2873634

[B29] Lindau D. Mussard J. Wagner B. J. Ribon M. Rönnefarth V. M. Quettier M.. (2013). Primary Blood Neutrophils Express a Functional Cell Surface Toll-Like Receptor 9. Eur. J. Immunol. 43, 2101–2113. 10.1002/eji.201142143 23686399

[B30] Liu L. Mao Y. Xu B. Zhang X. Fang C. Ma Y.. (2019). Induction of Neutrophil Extracellular Traps During Tissue Injury: Involvement of STING and Toll-like Receptor 9 Pathways. Cell Prolif. 52, 1–12. 10.1111/cpr.12579 PMC6536408 30851061

[B31] Lupetti A. Danesi R. Campa M. Tacca M.D. Kelly S. (2002). Molecular Basis of Resistance to Azole Antifungals. Trends Mol. Med. 8, 76–81. 10.1016/S1471-4914(02)02280-3 11815273

[B32] Manfredi A. A. Ramirez G. A. Rovere-Querini P. Maugeri N. (2018). The Neutrophil’s Choice: Phagocytose vs Make Neutrophil Extracellular Traps. Front. Immunol. 9:288. 10.3389/fimmu.2018.00288 29515586 PMC5826238

[B33] Martins M. Henriques M. Lopez-Ribot J. L. Oliveira R. (2012). Addition of DNase Improves the In Vitro Activity of Antifungal Drugs Against *Candida Albicans* Biofilms. Mycoses 55, 80–85. 10.1111/j.1439-0507.2011.02047.x 21668524 PMC3175262

[B34] Martins M. Uppuluri P. Thomas D. P. Cleary I. A. Henriques M. Lopez-Ribot J. L.. (2010). Presence of Extracellular DNA in the *Candida Albicans* Biofilm Matrix and its Contribution to Biofilms. Mycopathologia 169, 323–331. 10.1007/s11046-009-9264-y 20012895 PMC3973130

[B35] Mayer F. L. Wilson D. Hube B. (2013). *Candida Albicans* Pathogenicity. Virulence 4, 119–128. 10.4161/viru.22913 23302789 PMC3654610

[B36] Molin S. Tolker-Nielsen T. (2003). Gene Transfer Occurs With Enhanced Efficiency in Biofilms and Induces Enhanced Stabilisation of the Biofilm Structure. Curr. Opin. Biotechnol. 14, 255–261. 10.1016/S0958-1669(03)00036-3 12849777

[B37] Mulcahy H. Charron-Mazenod L. Lewenza S. (2008). Extracellular DNA Chelates Cations and Induces Antibiotic Resistance in *Pseudomonas Aeruginosa* Biofilms. PLoS Pathog. 4, 1–17. 10.1371/journal.ppat.1000213 PMC2581603 19023416

[B38] Naglik J. R. Gaffen S. L. Hube B. (2019). Candidalysin: Discovery and Function in *Candida Albicans* Infections. Curr. Opin. Microbiol. 52, 100–109. 10.1016/j.mib.2019.06.002 31288097 PMC6687503

[B39] Naglik J. R. Moyes D. Makwana J. Kanzaria P. Tsichlaki E. Weindl G.. (2008). Quantitative Expression of the *Candida Albicans* Secreted Aspartyl Proteinase Gene Family in Human Oral and Vaginal Candidiasis. Microbiology 154, 3266–3280. 10.1099/mic.0.2008/022293-0.Quantitative 18957581 PMC2722715

[B40] Nascimento M. Gombault A. Lacerda-Queiroz N. Panek C. Savigny F. Sbeity M.. (2019). Self-DNA Release and STING-dependent Sensing Drives Inflammation to Cigarette Smoke in Mice. Sci. Rep. 9, 1–10. 10.1038/s41598-019-51427-y 31619733 PMC6795997

[B41] Nobile C. J. Nett J. E. Andes D. R. Mitchell A. P. (2006). Function of *Candida Albicans* Adhesin Hwp1 in Biofilm Formation. Eukaryot. Cell 5, 1604–1610. 10.1128/EC.00194-06 17030992 PMC1595337

[B42] Oh K. B. Miyazawa H. Naito T. Matsuoka H. (2001). Purification and Characterization of an Autoregulatory Substance Capable of Regulating the Morphological Transition in *Candida Albicans* . Proc. Natl. Acad. Sci. U. S. A. 98, 4664–4668. 10.1073/pnas.071404698 11274356 PMC31891

[B43] Okshevsky M. Meyer R. L. (2015). The Role of Extracellular DNA in the Establishment, Maintenance and Perpetuation of Bacterial Biofilms. Crit. Rev. Microbiol. 41, 341–352. 10.3109/1040841X.2013.841639 24303798

[B44] Pilsczek F. H. Salina D. Poon K. K. H. Fahey C. Yipp B. G. Sibley C. D.. (2010). A Novel Mechanism of Rapid Nuclear Neutrophil Extracellular Trap Formation in Response to *Staphylococcus Aureus* . J. Immunol. 185, 7413–7425. 10.4049/jimmunol.1000675 21098229

[B45] Rajendran R. Sherry L. Lappin D. F. Nile C. J. Smith K. Williams C.. (2014). Extracellular DNA Release Confers Heterogeneity in *Candida Albicans* Biofilm Formation. BMC Microbiol. 14, 1–9. 10.1186/s12866-014-0303-6 PMC4262977 25476750

[B46] Rapala-Kozik M. Bochenska O. Zajac D. Karkowska-Kuleta J. Gogol M. Zawrotniak M.. (2018). Extracellular Proteinases of *Candida Species* Pathogenic Yeasts. Mol. Oral. Microbiol. 33, 113–124. 10.1111/omi.12206 29139623

[B47] Rochael N. C. Guimarães-Costa A. B. Nascimento M. T. C. DeSouza-Vieira T. S. Oliveira M. P. Garcia e Souza L. F.. (2015). Classical ROS-dependent and Early/Rapid ROS-Independent Release of Neutrophil Extracellular Traps Triggered by *Leishmania Parasites* . Sci. Rep. 5, 18302. 10.1038/srep18302 26673780 PMC4682131

[B48] Rodriguez-Rodrigues N. Castillo L. A. Landoni V. I. Martire-Greco D. (2017). Prokaryotic RNA Associated to Bacterial Viability Induces Polymorphonuclear Neutrophil Activation. Front. Cell. Infect. Microbiol. 7, 306. 10.3389/fcimb.2017.00306 28730145 PMC5498479

[B49] Rostami N. Shields R. C. Yassin S. A. Hawkins A. R. Bowen L. Luo T. L.. (2016). A Critical Role for Extracellular DNA in Dental Plaque Formation. J. Dent. Res. 96, 208–216. 10.1177/0022034516675849 27770039

[B50] Saitoh T. Komano J. Saitoh Y. Misawa T. Takahama M. Kozaki T.. (2012). Neutrophil Extracellular Traps Mediate a Host Defense Response to Human Immunodeficiency Virus-1. Cell Host Microbe 12, 109–116. 10.1016/j.chom.2012.05.015 22817992

[B51] Sapaar B. Nur A. Hirota K. Yumoto H. Murakami K. Amoh T.. (2014). Effects of Extracellular DNA From *Candida Albicans* and Pneumonia-Related Pathogens on *Candida* Biofilm Formation and Hyphal Transformation. J. Appl. Microbiol. 116, 1531–1542. 10.1111/jam.12483 24661775

[B52] Saville S. P. Lazzell A. L. Monteagudo C. Lopez-Ribot J. L. (2003). Engineered Control of Cell Morphology In Vivo Reveals Distinct Roles for Yeast and Filamentous Forms of *Candida Albicans* During Infection. Eukaryot. Cell 2, 1053–1060. 10.1128/EC.2.5.1053 14555488 PMC219382

[B53] Seviour T. W. Winnerdy F. R. Wong L. L. Shi X. Mugunthan S. Castaing R.. (2019). The Biofilm Matrix Scaffold of Pseudomonas Species Contains Non-Canonically Base Paired Extracellular DNA and RNA. Biochemistry 10.1101/527267 PMC7979868 33741996

[B54] Singel K. L. Grzankowski K. S. Khan ANMNH Grimm M. J. D’Auria A. C. Morrell K.. (2019). Mitochondrial DNA in the Tumour Microenvironment Activates Neutrophils and is Associated With Worse Outcomes in Patients With Advanced Epithelial Ovarian Cancer. Br. J. Cancer 120, 207–217. 10.1038/s41416-018-0339-8 30518816 PMC6342981

[B55] Soll D. R. Galask R. Schmid J. A. N. Hanna C. Mac K. Morrow B. (1991). Genetic Dissimilarity of Commensal Strains of Candida Spp. Carried in Different Anatomical Locations of the Same Healthy Women. J. Clin. Microbiol. 29, 1702–1710. 10.1128/JCM.29.8.1702-1710.1991 1761692 PMC270187

[B56] Taff H. T. Mitchell K. F. Edward J. A. Andes D. R. (2013). Mechanisms of *Candida* Biofilm Drug Resistance. Future Microbiol. 8, 1325–1337. 10.2217/fmb.13.101 24059922 PMC3859465

[B57] Thewes S. Moran G. P. Magee B. B. Schaller M. Sullivan D. J. Hube B. (2008). Phenotypic Screening, Transcriptional Profiling, and Comparative Genomic Analysis of an Invasive and non-Invasive Strain of *Candida Albicans* . BMC Microbiol. 8, 1–16. 10.1186/1471-2180-8-187 18950481 PMC2579918

[B58] Urban C. F. Ermert D. Schmid M. Abu-Abed U. Goosmann C. Nacken W.. (2009). Neutrophil Extracellular Traps Contain Calprotectin, a Cytosolic Protein Complex Involved in Host Defense Against *Candida Albicans* . PLoS Pathog. 5, e1000639. 10.1371/journal.ppat.1000639 19876394 PMC2763347

[B59] Urban C. F. Reichard U. Brinkmann V. Zychlinsky A. (2006). Neutrophil Extracellular Traps Capture and Kill *Candida Albicans* and Hyphal Forms. Cell. Microbiol. 8, 668–676. 10.1111/j.1462-5822.2005.00659.x 16548892

[B60] Whitchurch C. B. Tolker-nielsen T. Ragas P. C. John S. Whitchurch C. B. Tolker-Nielsen T.. (2002). Extracellular DNA Required for Bacterial Biofilm Formation. Science 295 (5559), 1487. 10.1126/science.295.5559.1487 11859186

[B61] Zarnowski R. Westler W. M. Lacmbouh G. A. Marita J. M. Bothe J. R. Bernhardt J.. (2014). Novel Entries in a Fungal Biofilm Matrix Encyclopedia. MBio 5, 1–13. 10.1128/mBio.01333-14 PMC4128356 25096878

[B62] Zawrotniak M. Bochenska O. Karkowska-Kuleta J. Seweryn-Ozog K. Aoki W. Ueda M.. (2017). Aspartic Proteases and Major Cell Wall Components in Candida Albicans Trigger the Release of Neutrophil Extracellular Traps. Front. Cell. Infect. Microbiol. 7, 414. 10.3389/fcimb.2017.00414 28983472 PMC5613151

[B63] Zawrotniak M. Wojtalik K. Rapala-Kozik M. (2019). Farnesol, a Quorum-Sensing Molecule of Candida Albicans Triggers the Release of Neutrophil Extracellular Traps. Cells 8, 1611. 10.3390/cells8121611 PMC6952927 31835824

[B64] Zhang S. Hu Z. Tanji H. Jiang S. Das N. Li J.. (2018). Small-Molecule Inhibition of TLR8 Through Stabilization of Its Resting State. Nat. Chem. Biol. 14, 58–64. 10.1038/nchembio.2518 29155428 PMC5726935

