# Supplementary material for: Extracellular Nucleic Acids Present in the Candida albicans Biofilm Trigger the Release of Neutrophil Extracellular Traps
Source: Front Cell Infect Microbiol. 2021 May 26;11:681030. doi: 10.3389/fcimb.2021.681030 (PMC8187917; doi:10.3389/fcimb.2021.681030)
Supplement: Supplementary file 1 [file Table_1.docx]

Supplementary Material


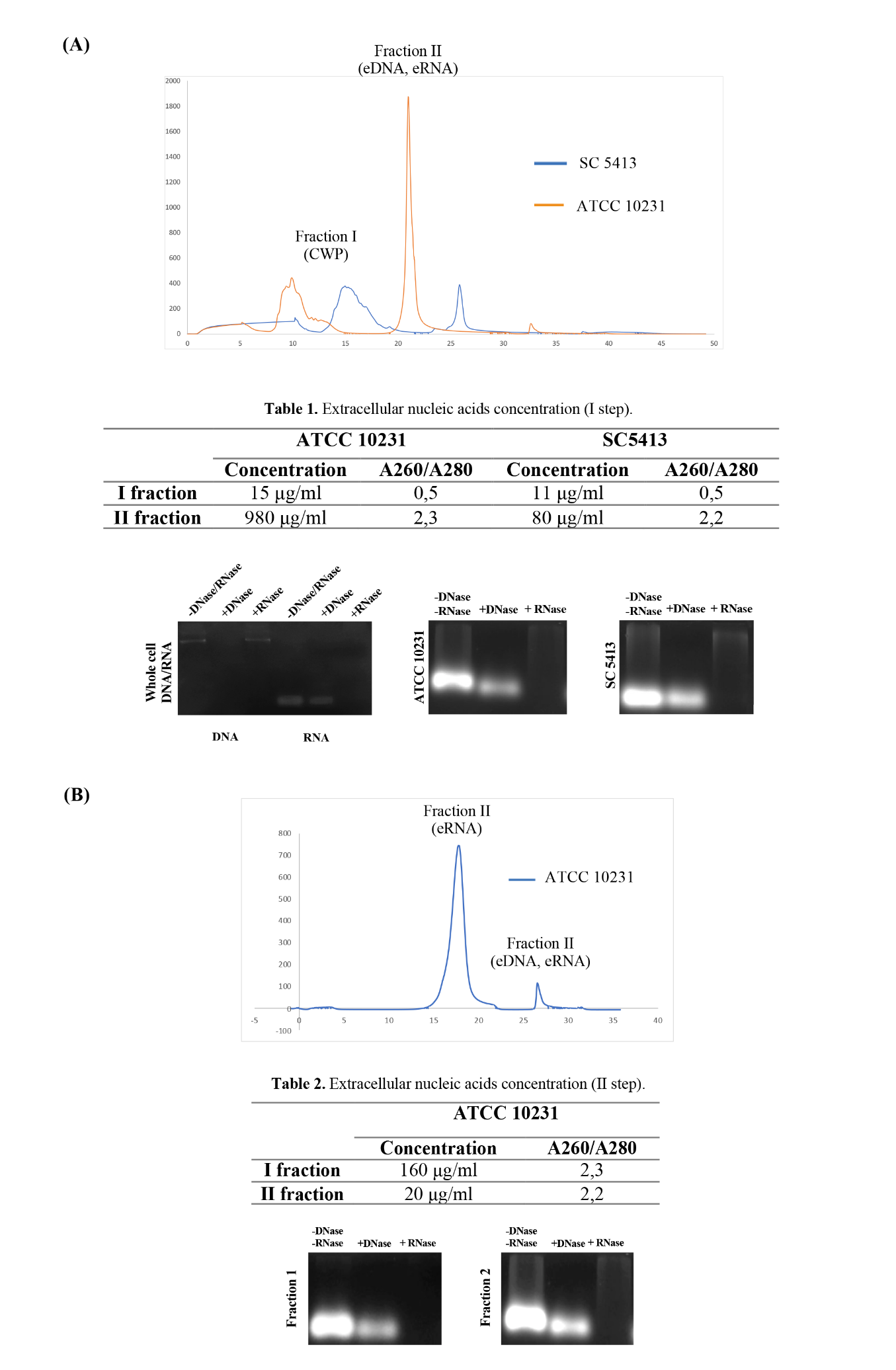


**Supplementary Figure 1. Purification of extracellular nucleic acids from biofilm ECM using IEC.** *C. albicans* biofilms (strains ATCC 10231 and SC5413) were grown at 37°C for 48 hours in Erlenmeyer flasks. Then the ECM isolation, based on the enzymatic degradation of the yeast cell wall by β-1,3-glucanase (1250 U per 1 g of biofilm wet mass) was performed. **(A)** Nucleic acids were separated from the remaining compounds on MonoQ HR 10/10 column, using a 10 min NaCl gradient (0-0.4M; fraction I) with 10 min isocratic elution and 10 min gradient (0.4-1M; fraction II) in 50 mM Tris-HCl buffer (pH 7.5-8.5). The nucleic acid concentration was measured spectrophotometrically at 260 nm, using Biotek Synergy H1 microplate reader (Table 1). Collected samples of extracellular nucleic acids (fraction II) for both strains were analyzed electrophoretically (1% agarose gel) without and after DNase I and RNase A treatment for 30 min. As a control, the same treatment was performed for the yeast genomic DNA and total RNA isolated from yeast cells. **(B)** The fractions containing the highest concentration of nucleic acids were dialyzed against 20 mM MOPS buffer with 350 mM NaCl (pH 6.25) and the second chromatographic step on the MonoQ column was applied. In th elution process NaCl gradient (0,3 – 1M) in 20 mM MOPS (pH 6.25) was used (data presented for strain ATCC 10231). The concentration and purity of the nucleic acids were assessed electropherically and spectrophotometrically (Table 2) as descbribe above.


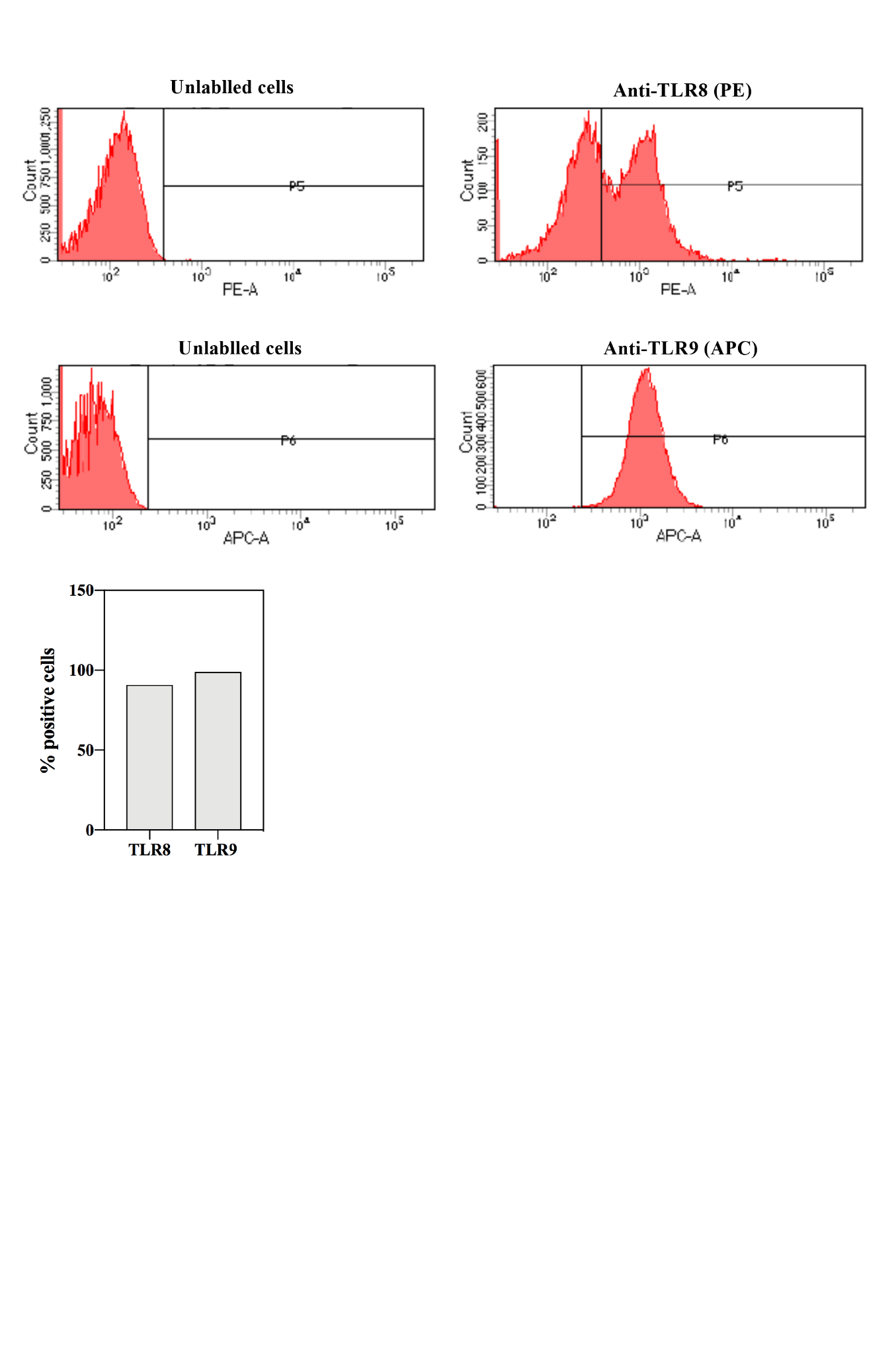


**Supplementary Figure 2. Identification of TLR8 and TLR9 expression in human neutrophils.** Freshly isolated neutrophils were fixed with 3.6% paraformaldehyde, then, the cells were permeabilized with Triton X-100, and after washing twice with sterile PBS, the cells were overnight incubated at 4°C with phycoerythrin-conjugated (PE) anti-human TLR8 antibodies (1:200; BioLegend) and allophycocyanin-conjugated (APC) ant-human TLR9 (1:200; BioLegend). Data present the result obtained by BD LSR Fortressa flow cytometr.

**
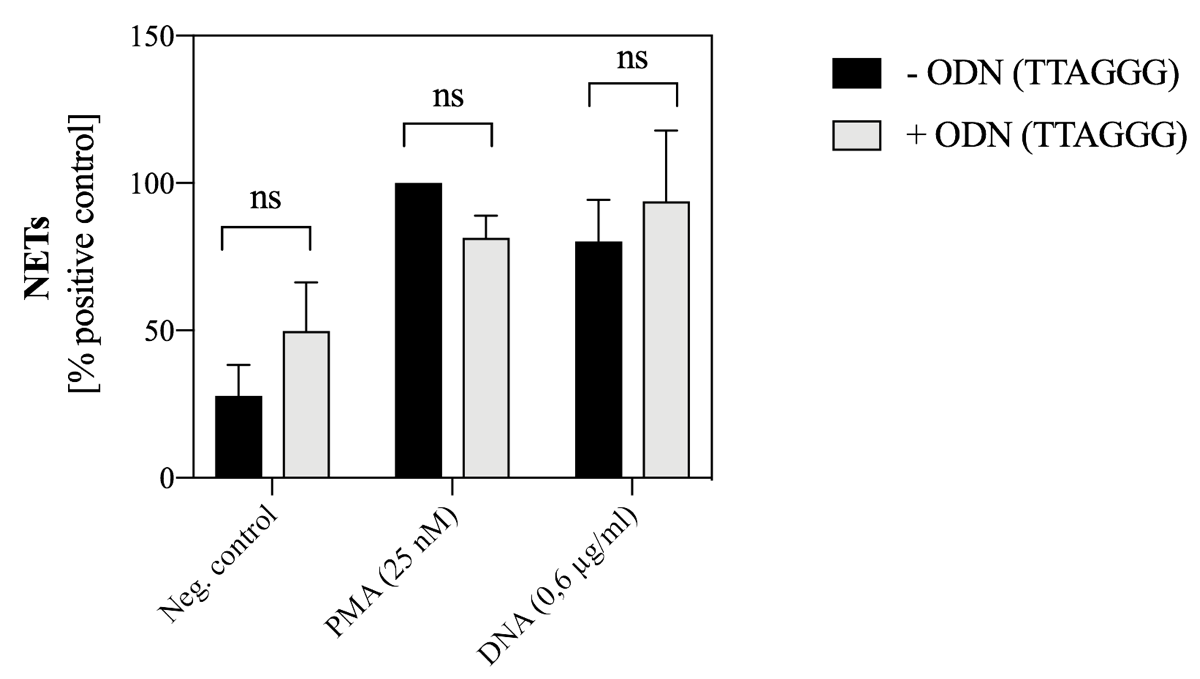
**

**Supplementary Figure 3. The involvement of TLR9 receptor in netosis activation by *C. albicans* DNA.** A. Neutrophils (10^6^ cells/ml; 100 μl per well) were stimulated at 37°C in 5% CO_2_ atmosphere for 3 h with yeast DNA (0,6 μg/ml) or PMA (positive control; final concentration 25 nM) diluted in RPMI-1640 medium containing CpGODN solution (100 nM). RPMI-1640 medium with the same concentration of CpGODN served as the negative control. Released NET was stained with Sytox Green (1 μM) and quantified in supernatants after MNase treatment (final concentration 1 U/ml). Differences between the netosis intensity were considered statistically insignificant (ns) for p> 0.05.
